# Mutations Causing Complex Disease May under Certain Circumstances Be Protective in an Epidemiological Sense

**DOI:** 10.1371/journal.pone.0132150

**Published:** 2015-07-10

**Authors:** Sabine Siegert, Andreas Wolf, David N. Cooper, Michael Krawczak, Michael Nothnagel

**Affiliations:** 1 Cologne Center for Genomics, University of Cologne, Cologne, Germany; 2 Institute of Epidemiology, Christian-Albrechts University, Kiel, Germany; 3 Institute of Medical Informatics and Statistics, Christian-Albrechts University, Kiel, Germany; 4 Institute of Medical Genetics, School of Medicine, Cardiff University, Cardiff, United Kingdom; Ohio State University Medical Center, UNITED STATES

## Abstract

Guided by the practice of classical epidemiology, research into the genetic basis of complex disease has usually taken for granted the dictum that causative mutations are invariably over-represented among clinically affected as compared to unaffected individuals. However, we show that this supposition is not true and that a mutation contributing to the etiology of a complex disease can, under certain circumstances, be depleted among patients. Populations with defined disease prevalence were repeatedly simulated under a Wright-Fisher model, assuming various types of population history and genotype-phenotype relationship. For each simulation, the resulting mutation-specific population frequencies and odds ratios (ORs) were evaluated. In addition, the relationship between mutation frequency and OR was studied using real data from the NIH GWAS catalogue of reported phenotype associations of single-nucleotide polymorphisms (SNPs). While rare diseases (prevalence <1%) were found to be consistently caused by rare mutations with ORs>1, up to 20% of mutations causing a pandemic disease (prevalence 10–20%) had ORs<1, and their population frequency ranged from 0% to 100%. Moreover, simulation-based ORs exhibited a wide distribution, irrespective of mutation frequency. In conclusion, a substantial proportion of mutations causing common complex diseases may appear ‘protective’ in genetic epidemiological studies and hence would normally tend to be excluded, albeit erroneously, from further study. This apparently paradoxical result is explicable in terms of mutual confounding of the respective genotype-phenotype relationships due to a negative correlation between causal mutations induced by their common gene genealogy. As would be predicted by our findings, a significant negative correlation became apparent in published genome-wide association studies between the OR of genetic variants associated with a particular disease and the prevalence of that disease.

## Introduction

In the past decade, a large number of genome-wide association studies (GWAS) have been undertaken in order to dissect the genetic basis of complex diseases [1,2]. Indeed, since the first such study was published in 2005 [3], more than 1200 susceptibility loci for >165 common human diseases have been identified by GWAS [4–6]. Although most of the genotype-phenotype associations reported from GWAS were modest at best, even if successfully replicated [4,7], and often involved variants located outside functionally relevant gene regions, these studies have nevertheless provided important new insights into the etiology of complex human diseases [4,8]. This notwithstanding, genetic risk factors hitherto identified by GWAS usually account for only a small fraction of the heritability of the disease in question. Indeed, for the vast majority of conditions studied, >90% of the population-level phenotypic variation has remained unaccounted for by reference to any known genetic variation [6,9]. Several explanations for this outcome have been put forward, including a major role for rare as yet unidentified variants, structural features such as copy-number variation (CNVs), power loss due to excessive multiple testing, locus and allelic heterogeneity, parent of origin effects and gene-gene as well as gene-environment interactions [4,5,10,11].

In addition, a number of sometimes counterintuitive phenomena have been described to occur in the context of genetic disease associations. For example, risk alleles often change their role from predisposing to protective and *vice versa* in different populations, thereby spawning so-called ‘flip-flop associations’ that probably reflect gene-gene interaction and differential linkage disequilibrium [12]. Moreover, a ‘synthetic association’ [13] between a disease and a common non-causative variant can arise as a consequence of the chance accumulation of several rare causative variants *in cis* to the latter [14–16]. Finally, it has been demonstrated by simulation that, in multi-locus systems of correlated causal and non-causal genetic factors, an ‘indirect association’ between a non-causal variant and a disease can arise even in the absence of any causative genes in the vicinity [17].

**Fig 1:**
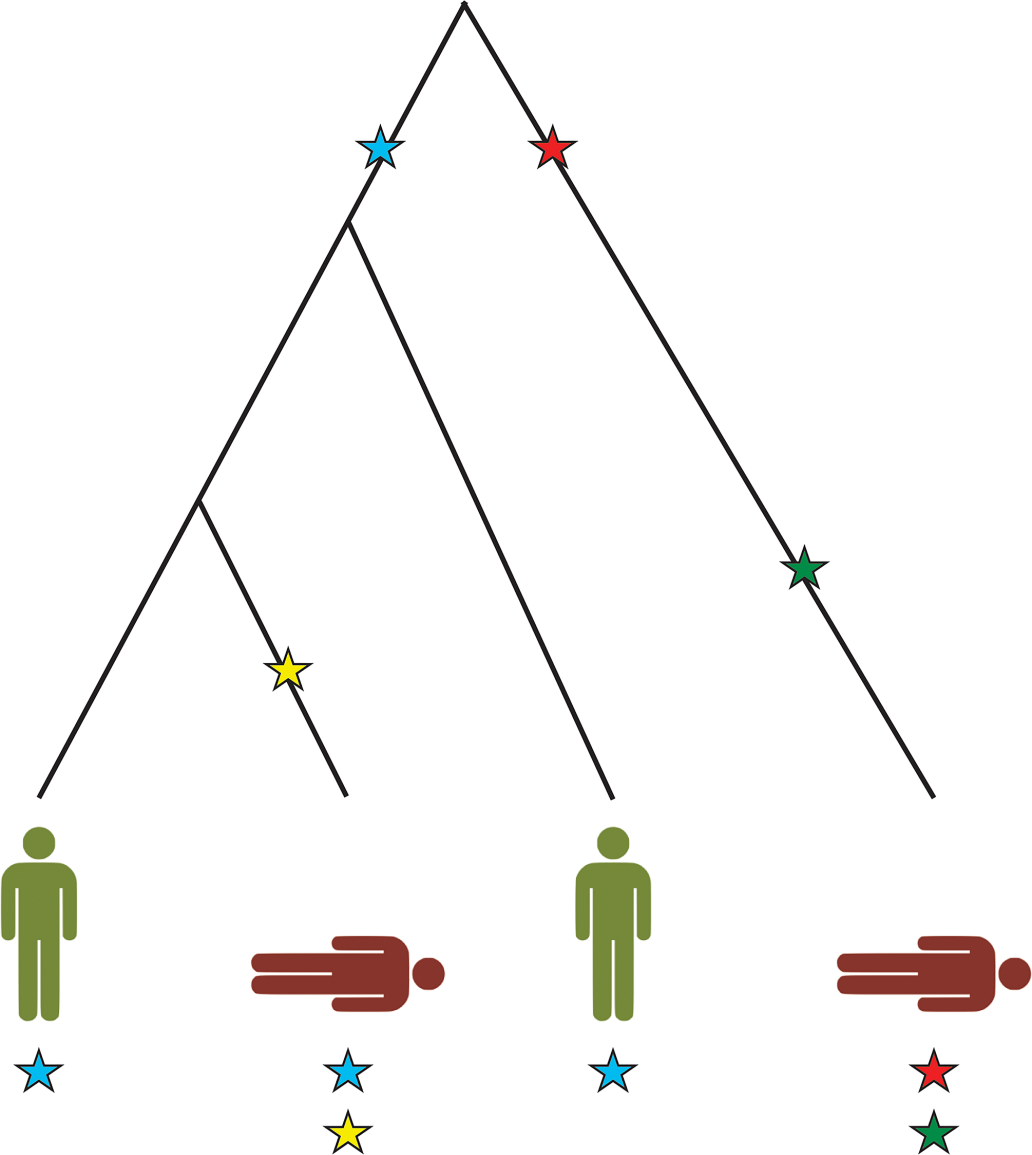
Illustration of a genealogy-actuated negative correlation between causative mutations. The underlying penetrance model assumes that two or more causative mutations are required for an individual to become affected (with certainty). The blue mutation is depleted among affected individuals in this population of four and, therefore, is protective in an epidemiological sense. The other mutations are enriched in cases.

Here, we report a population history mechanism that may obfuscate the causal relationship between a genetic variant and a (common) human disease. In classical epidemiology, making inference about disease causation is usually equivalent to ascertaining whether or not a given exposure confers a relative risk greater than unity. However, this process of reasoning (also enshrined in the concept of ‘attributable fractions’ [18]) may be confounded by other unmeasured causal factors. In particular, a causative exposure may pose as a protective factor rather than a risk factor if it is negatively correlated with its causal complement, defined as those other risk factors or combinations of risk factors that are both necessary and sufficient for the exposure in question to cause disease [18]. In genetic epidemiology, genotypes play the role of risk factors and, since many human diseases follow a complex oligogenic or polygenic mode of inheritance [19], causal complementation is probably omnipresent (see Fig 1). Moreover, all humans share a common genealogy which induces varying levels of inter- and intra-individual dependency between genotypes [20].

In view of the above, any uncritical adoption of the paradigm from classical epidemiology that disease-causing genetic variants should invariably be over-represented in the clinically affected portion of the population under study (i.e. that they confer a relative disease risk exceeding unity) [21] may be misleading. In fact, we show by simulation that, depending upon the specifics of the genealogy of the population of interest, a causative mutation may well appear “protective” in an epidemiological sense. Normally, the functional follow-up of such a variant would end up being inconclusive simply because its damaging character would be difficult to reconcile with its protective effect. In many instances, the variant would then be erroneously excluded from further consideration. In order to lend additional support to our conclusions, we also present empirical evidence for the presence, in real GWAS, of an inverse relationship between epidemiological effect size and disease prevalence as would be predicted by the above mechanism of confounding.

## Materials and Methods

### Simulations

We investigated various models of population history and penetrance to determine the impact upon epidemiological effect size of a genealogy-related negative correlation between disease-causing mutations. To this end, we repeatedly simulated the genealogy of a population of constant size (*N* = 10,000) following a coalescent approach [22]. The coalescent is a well-established means of modeling population histories which, compared to forward-time simulation, is both easier to implement and computationally more efficient [23]. We initially adopted a Wright-Fisher model [24] of a single haploid locus was initially adopted in order to model the evolution of functionally relevant variation within genes or gene regions small enough to escape internal meiotic recombination. Subsequently, we extended our simulations to oligogenic models of inheritance involving 2, 5 or 10 unlinked and hence independently evolving loci in order to assess the possible influence of recombination on our results as well. After the simulation of each locus-specific genealogy, causative mutations were randomly placed on the branches of each coalescence tree with probabilities proportional to the respective branch lengths.

**Fig 2:**
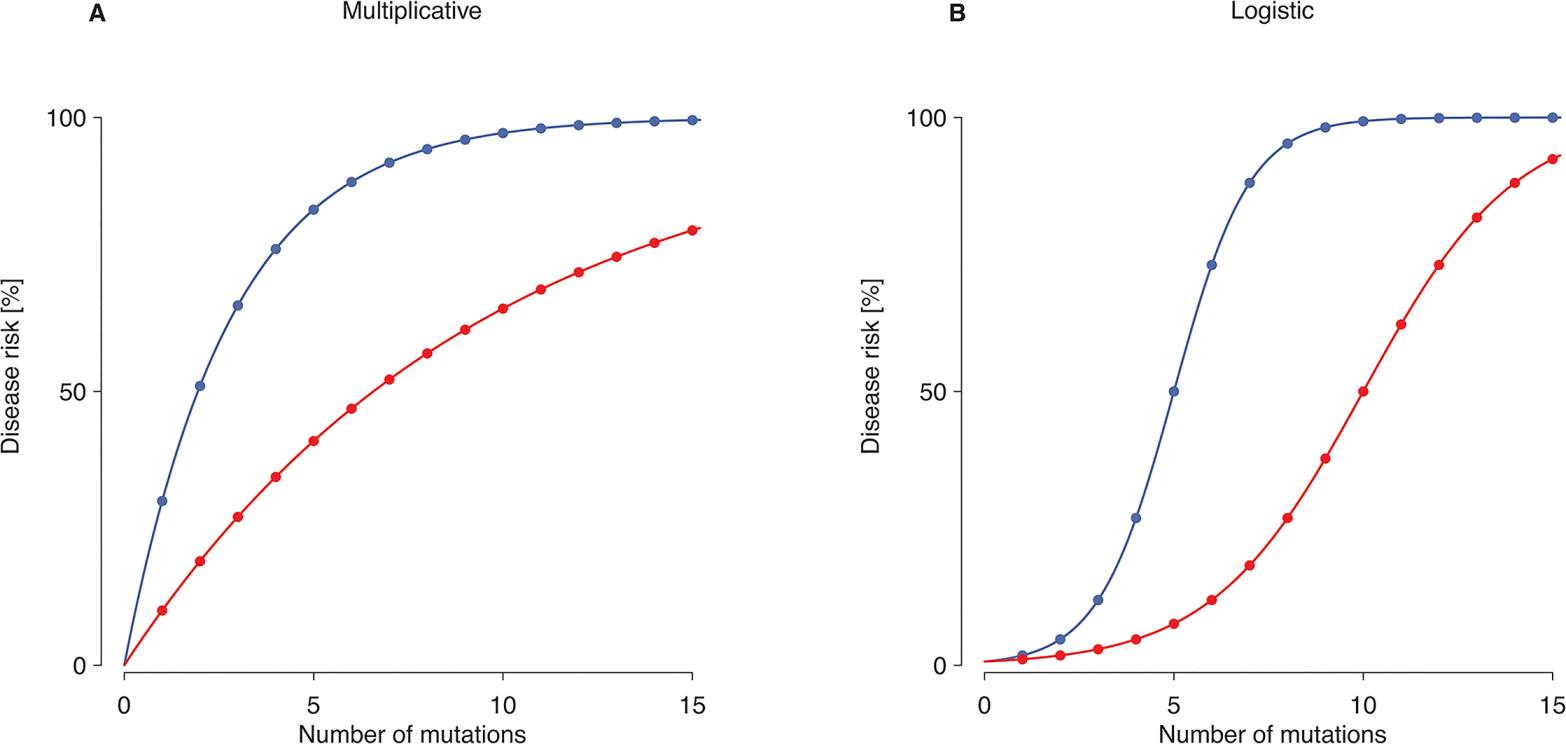
Disease risk as a function of the number of mutations present under two different penetrance models. (A) Multiplicative model with parameter γ = 0.3 (blue) or γ = 0.1 (red). (B) Logistic model with parameters *α* = -5; *β* = 1 (blue) or *α* = -5; *β* = 0.5 (red).

Once all locus-specific trees and the distribution of mutations on the trees had been determined in a given simulation, each leaf (i.e. haploid individual) was randomly assigned a dichotomous disease state with probability *P*(*k*), where *k* denotes the number of causative mutations present at all loci combined. We considered two biologically plausible penetrance models that have been widely used for similar analyses before [25–27]. More specifically, we used a one-parameter multiplicative model (Fig 2A),

$$P\left(k\right)=1-{\left(1-\gamma \right)}^{k}\;\text{with}\;0<\gamma <1,$$ (1)

which implies a steep increase in disease probability with increasing mutation number, and a two-parameter logistic model (Fig 2B),

logit[P(k)]=α+β∙kwithβ>0, (2)

invoking tolerance against a small number of mutations. Both the multiplicative model and the logistic model assume that mutations exert their additive effects on the respective scale. However, whereas individuals lacking a mutation (i.e. *k* = 0) would be unaffected under the multiplicative model, their (baseline) risk equals 1/(1+*e* ^-*α*^)>0 under the logistic model. For both models, we chose parameter values inducing either a strong or a weak increase in disease probability with mutation number.

We also modeled oligogenic disease etiologies that involved 2, 5 or 10 unlinked loci (e.g. genes on different chromosomes), respectively. Since unlinked loci evolve essentially independently in outbred populations, they also have nearly independent genealogies [28]. For this reason, we repeatedly simulated the unrelated history of 2, 5 or 10 haploid loci, respectively, and randomly assigned each individual a dichotomous disease state with probability *P*({*k*_1_,…,*k*_*m*_}), where *k*_*i*_ denotes the number of causative mutations at the *i-th* locus. Again, we considered both a multiplicative model,

$$P\left(\left\{k_{1},\ldots ,k_{m}\right\}\right)=1-{\left(1-\gamma \right)}^{\sum_{i=1}^{m}k_{i}}\;\text{with}\;0<\gamma <1$$ (3)

and a logistic model,

$$\text{logit}\left[P\left(\left\{k_{1},\ldots ,k_{m}\right\}\right)\right]=\alpha +\beta ∙\sum_{i=1}^{m}k_{i}\;\text{with}\;\beta >0,$$ (4)

both of which assume the same contribution to the disease risk by all *m* loci. Only those simulations with disease prevalence within a pre-specified range were selected for further analysis. Simulations were repeatedly performed until 1,000 such datasets had been obtained per prevalence range. For each dataset, the mutation-specific population frequencies and odds ratios (ORs) were calculated, except for those mutations that were exclusively present in either cases or controls. Simulations were carried out with in-house scripts for Perl and R (S1 File). We used R v3.0.3 [29] for statistical analysis and for creating graphs.

### Analysis of GWAS data

In order to investigate whether the results of our inevitably simplifying simulations were consistent with real data, we scrutinized the phenotype associations of single-nucleotide polymorphisms (SNPs) compiled in the NIH GWAS catalogue [30] (http://www.genome.gov/26525384). Of the 14,869 records logged as of 13 November 2013, some 3,479 contained complete information on the trait-associated SNP allele, the minor allele frequency (MAF), OR and statistical significance (i.e. the p value). We merged reports with synonymous trait names (S1 Table) and, in order to improve data quality, we considered further only those traits for which at least 10 (not necessarily different) significant SNP associations (p<5×10^−8^) had been reported. Only traits of known prevalence in adults (>18 years) were included in our analysis. We adopted two different significance thresholds for our analysis, namely (i) p<5×10^−4^ in at least one GWAS to cover SNPs with small MAF and consequently low detection power, and (ii) p<5×10^−8^ which is generally deemed to constitute genome-wide significance. In order to minimize reporting bias, we initially considered only traits with prevalence between 0.1% and 20%. Later, we also included traits with prevalence <0.1% or adopted a maximum prevalence of 15% (S2 Table). We used the incidence rate as an approximation for prevalence in the case of the ‘sudden cardiac arrest’ trait. ORs were consistently considered to refer to the minor allele. Subsequently, we confined our analysis to those associations for which the derived allele of the involved SNP could be identified unambiguously, thereby accounting for the fact that all deleterious mutations in our simulations were introduced anew (S2 Table). The necessary ancestral allele information was retrieved from dbSNP (ftp://ftp.ncbi.nlm.nih.gov/snp/database/; downloaded May 27, 2015). In addition, we chose to consider only SNPs with unambiguous allele assignment, i.e. SNPs with allele combinations A/T and C/G were excluded. Linear regression analysis of the trait-specific OR (or median ORs) and the trait prevalence was carried out with R v3.0.3 and the statistical significance of non-zero regression coefficients assessed using a Wald test.

## Results

As in previous studies [25–27], we employed simplified models of population history for the coalescent simulation [22] and considered two types of combined penetrance, namely multiplicative or logistic. Although these two penetrance models are reminiscent of classical approaches to the study of discontinuous multifactorial traits, including Falconer’s threshold model [31], they are nevertheless different in that their affection probabilities are linked to mutation number in quantitative (i.e. via a mathematical function) rather than qualitative fashion (i.e. through a fixed threshold). Our analysis was stratified into three archetypal disease prevalence ranges, namely rare (0.1% to 1%), common (1% to 5%) and pandemic (5% to 20%). The simulations were carried out for both a single-locus and a two-locus model, where the latter was adopted to explore the possible effect of meiotic recombination on our conclusions.

**Fig 3:**
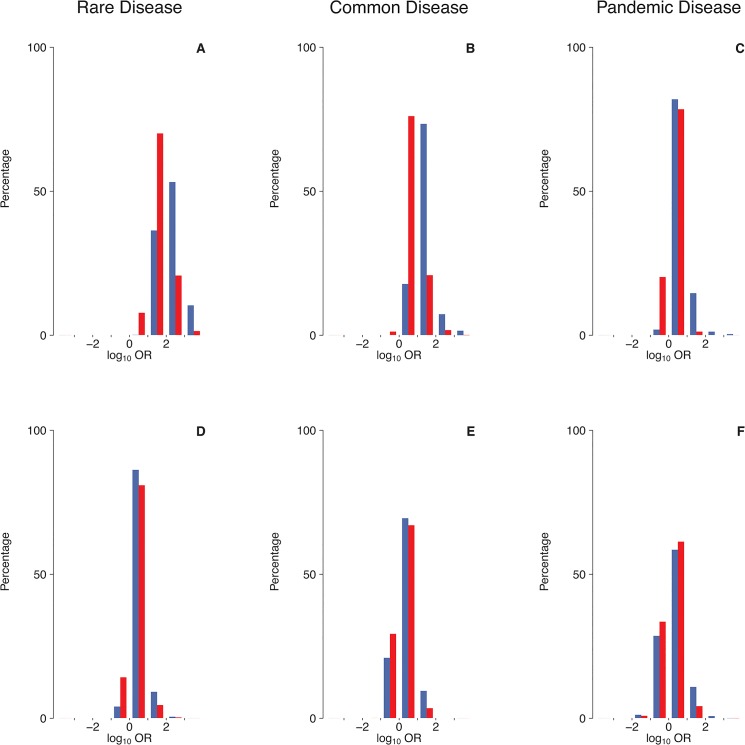
Epidemiological effect size of causative mutations at a single locus under two different penetrance models. The distribution of the log_10_ odds ratio (log_10_OR) is depicted for prevalence 0.1%-1.0% (A,D), 1%-5% (B,E) or 10%-20% (C,F), adopting either a multiplicative (A,B,C) or a logistic penetrance model (D,E,F). Blue: multiplicative model parameter γ = 0.3, logistic model parameters *α* = -5; *β* = 1; Red:γ = 0.1, *α* = -5; *β* = 0.5.

**Fig 4:**
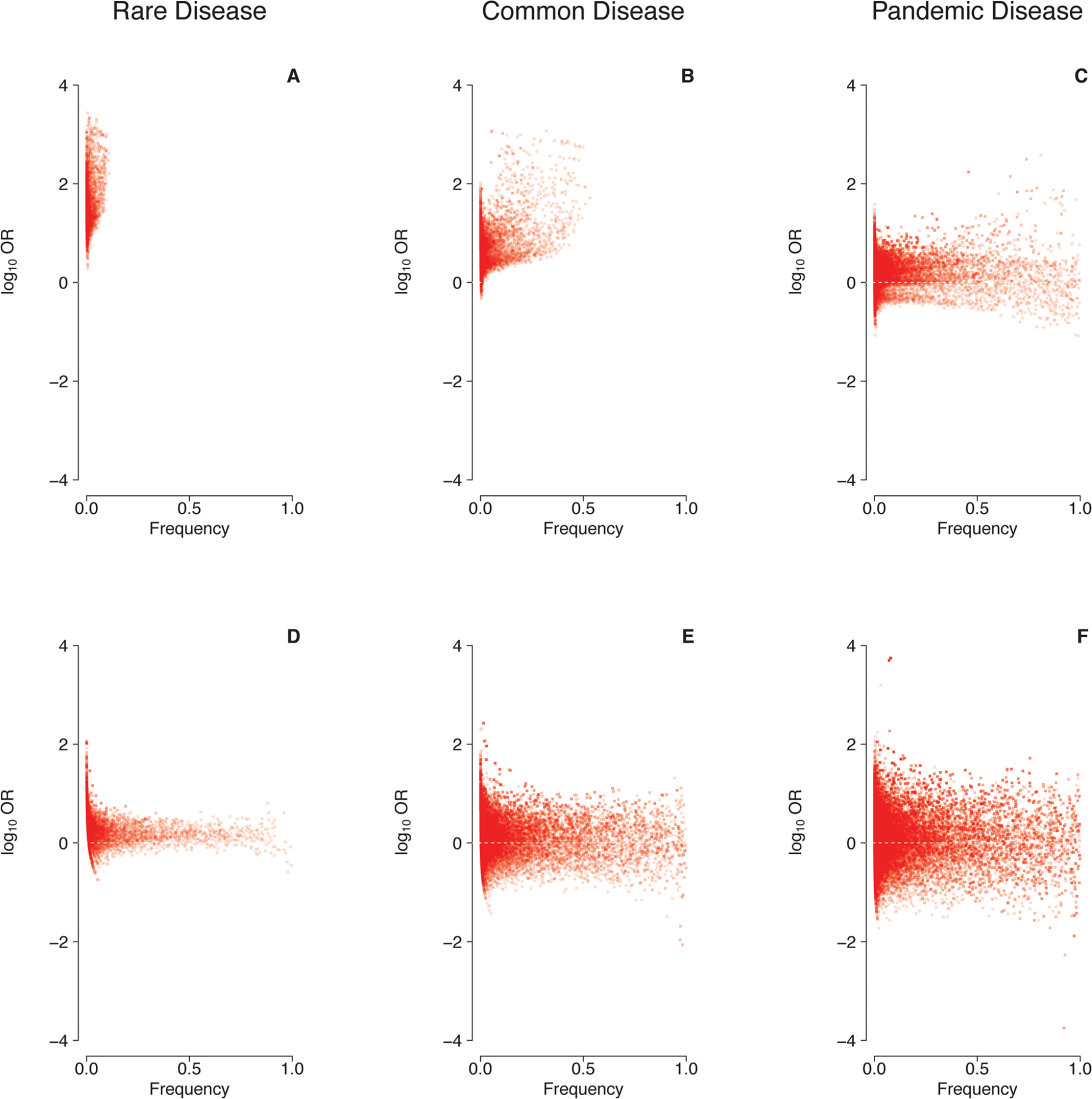
Relationship between epidemiological effect size (log_10_OR) and frequency of causative mutations at a single locus under two different penetrance models. Prevalence 0.1%-1.0% (A,D), 1%-5% (B,E) or 10–20% (C,F); multiplicative model with γ = 0.1 (A,B,C), logistic model with *α* = -5; *β* = 0.5 (D,E,F).

Under a multiplicative penetrance model precluding tolerance to a small number of mutations (Fig 2A), mutations at a single locus causing a rare disease (prevalence: 0.1-1.0%) were consistently found to have ORs greater than unity (Fig 3A), and to be themselves rare (Fig 4A). Virtually all mutations would be deemed to be disease-predisposing in this scenario because controls were rarely carriers (median number of mutations per control: 0.054 for γ = 0.3; 0.013 for γ = 0.1; Table 1). By contrast, mutations causing a common disease (prevalence: 1–5%) occasionally had OR<1 (up to 2.5% of simulations; Table 2 and Fig 3B), and many mutations occurred at a frequency >50% (Fig 4B). By contrast, for pandemic diseases (prevalence: 10–20%), a substantial proportion of causative mutations (up to 20% of simulations; Table 2) were characterized by OR<1 (Fig 3C), rendering these mutations “protective” in an epidemiological sense despite the lack of tolerance against a small number of causative mutations. Note that, even though several dozens of mutations may have been present in the population as a whole, the number of mutations per individual was consistently small (Table 1). The mutation frequencies were found to span the whole range between 0% and 100% (Fig 4C), and the median number of mutations present per case and present per control differed by less than threefold (Table 1).

**Table 1:** Spectrum of causative mutations at a single locus arising under two different penetrance models.

Model parameters	Median number of mutations (IQR)		
	Per population	Per case	Per control
**Rare disease (prevalence: 0.1–1%)**			
**Multiplicative**			
γ = 0.3	3 (2–5)	1.000 (1.000–1.000)	0.013 (0.007–0.019)
γ = 0.1	5 (3–7)	1.000 (1.000–1.000)	0.054 (0.030–0.076)
**Logistic**			
*α* = -5; *β* = 1	6 (3–9)	0.407 (0.202–0.552)	0.177 (0.086–0.264)
*α* = -5; *β* = 0.5	9 (5–15)	0.876 (0.678–1.051)	0.638 (0.467–0.805)
**Common disease (prevalence: 1–5%)**			
**Multiplicative**			
γ = 0.3	5 (4–8)	1.000 (1.000–1.045)	0.081 (0.049–0.103)
γ = 0.1	9 (5–13)	1.098 (1.006–1.370)	0.367 (0.254–0.434)
**Logistic**			
*α* = -5; *β* = 1	19 (13–28)	2.221 (1.996–2.449)	1.469 (1.267–1.655)
*α* = -5; *β* = 0.5	41 (29–56)	4.335 (4.079–4.608)	3.533 (3.262–3.746)
**Pandemic disease (prevalence: 10–20%)**			
**Multiplicative**			
γ = 0.3	11 (7–16)	1.241 (1.060–1.622)	0.442 (0.366–0.505)
γ = 0.1	24 (16–35)	2.391 (2.127–2.730)	1.877 (1.688–1.987)
**Logistic**			
*α* = -5; *β* = 1	37 (26–52)	3.979 (3.741–4.318)	2.836 (2.551–3.068)
*α* = -5; *β* = 0.5	74 (54–98)	7.617 (7.351–8.026)	6.346 (5.980–6.634)

IQR: inter-quartile range

**Table 2:** Proportion of epidemiologically protective mutations at a single locus under two different penetrance models.

Model parameters	Percentage mutations with OR<1.0	Percentage mutations with OR>*m* or OR<1/*m* (percentage of these with OR<1)		Percentage mutations with OR<1.0 among those with frequency <*f*		
		*m* = 1.5	*m* = 2.0	*f* = 0.001	*f* = 0.01	*f* = 0.05
**Rare disease (prevalence: 0.1–1%)**						
**Multiplicative**						
γ = 0.3	0.00	100.0 (0.00)	100.0 (0.00)	0.00	0.00	0.00
γ = 0.1	0.00	100.0 (0.00)	99.97 (0.00)	0.00	0.00	0.00
**Logistic**						
*α* = -5; *β* = 1	4.04	89.07 (1.29)	74.91 (0.50)	0.00	0.00	5.12
*α* = -5; *β* = 0.5	14.20	67.25 (6.57)	46.61 (3.69)	0.00	0.00	12.84
**Common disease (prevalence: 1–5%)**						
**Multiplicative**						
γ = 0.3	0.00	99.92 (0.00)	99.81 (0.00)	0.00	0.00	0.00
γ = 0.1	1.25	95.95 (0.23)	90.84 (0.03)	0.00	2.50	1.71
**Logistic**						
*α* = -5; *β* = 1	21.05	76.75 (15.53)	63.05 (12.06)	0.00	13.55	18.72
*α* = -5; *β* = 0.5	29.41	69.40 (23.25)	49.64 (18.86)	0.00	19.03	25.50
**Pandemic disease (prevalence: 10–20%)**						
**Multiplicative**						
γ = 0.3	1.94	93.51 (0.57)	83.49 (0.30)	4.15	3.66	2.59
γ = 0.1	20.23	67.16 (13.56)	46.35 (10.44)	12.12	20.92	20.44
**Logistic**						
*α* = -5; *β* = 1	29.81	80.58 (25.28)	64.11 (21.73)	9.60	24.21	27.07
*α* = -5; *β* = 0.5	34.38	72.01 (30.63)	54.37 (27.71)	12.57	29.32	32.19

OR: odds ratio

Under a logistic penetrance model invoking tolerance to a small number of mutations (Fig 2B), many more mutations than under a multiplicative model were required for the disease to occur (Table 1). Thus, the median number of mutations per case ranged from 6 for a rare disease (*α* = -5; *β* = 1) to 74 for a pandemic disease (*α* = -5; *β* = 0.5). At least for rare diseases, the discrepancy between the median numbers of mutations per case and per control was also much smaller than under a multiplicative model (Table 1), an effect that was mainly attributable to the non-zero baseline risk of the logistic model. In summary, the proportion of mutations with ORs <1 was found to be higher under a logistic model (up to 34%; Fig 3D–3F, Table 2) than under a multiplicative model for all prevalence ranges considered in our study, and ORs were more widely distributed irrespective of the individual mutation frequency (Fig 4D–4F).

**Fig 5:**
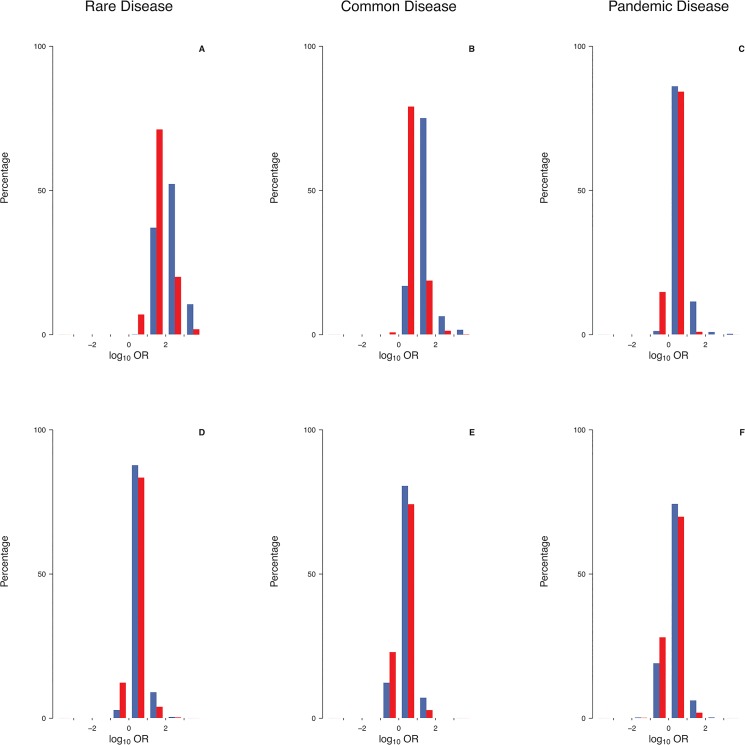
Epidemiological effect size of causative mutations at two unlinked loci under two different penetrance models. See legend to Fig 3 for details.

**Fig 6:**
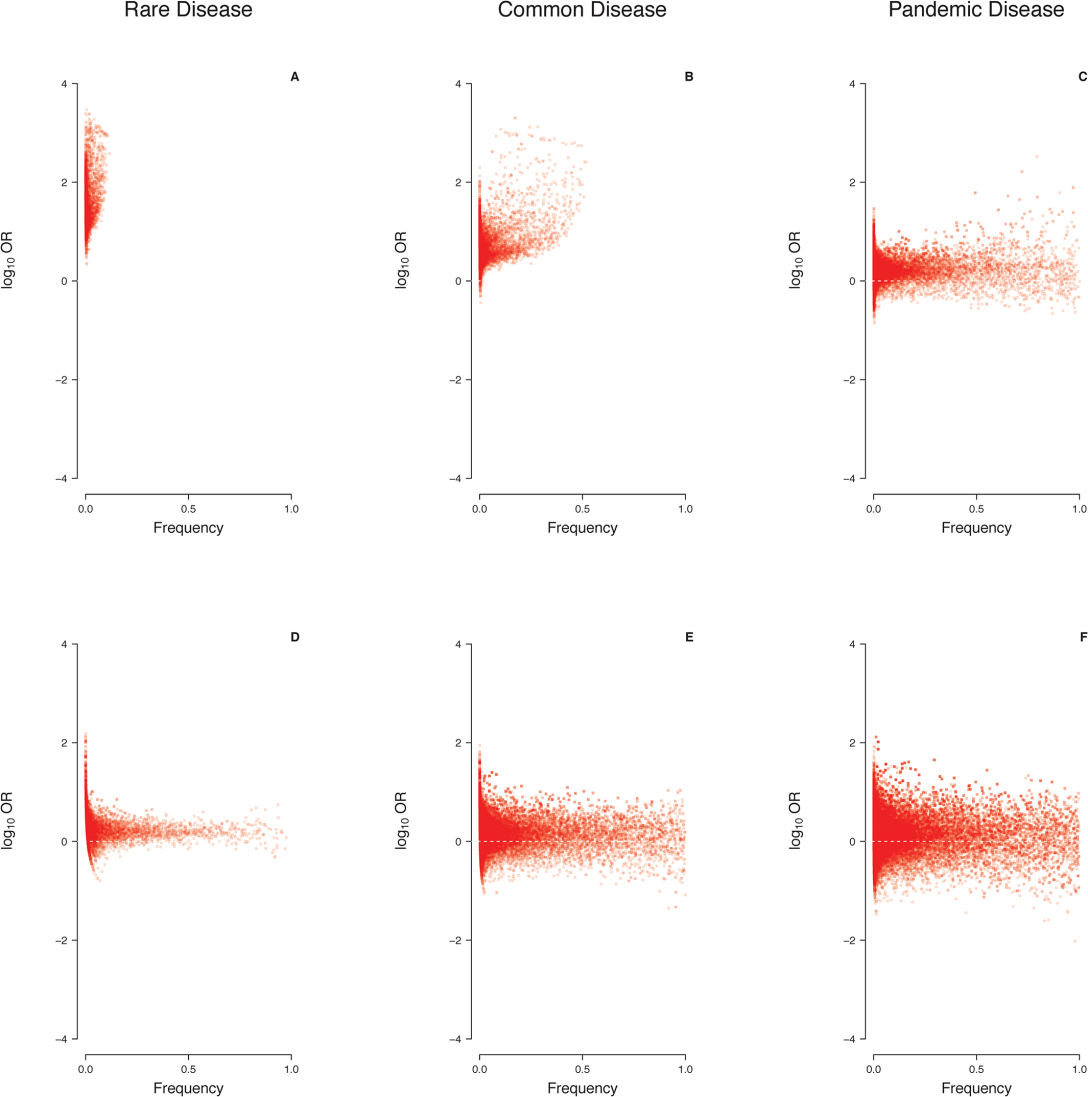
Relationship between epidemiological effect size (log_10_OR) and frequency of causative mutations at two unlinked loci under two different penetrance models. See legend to Fig 4 for details.

**Fig 7:**
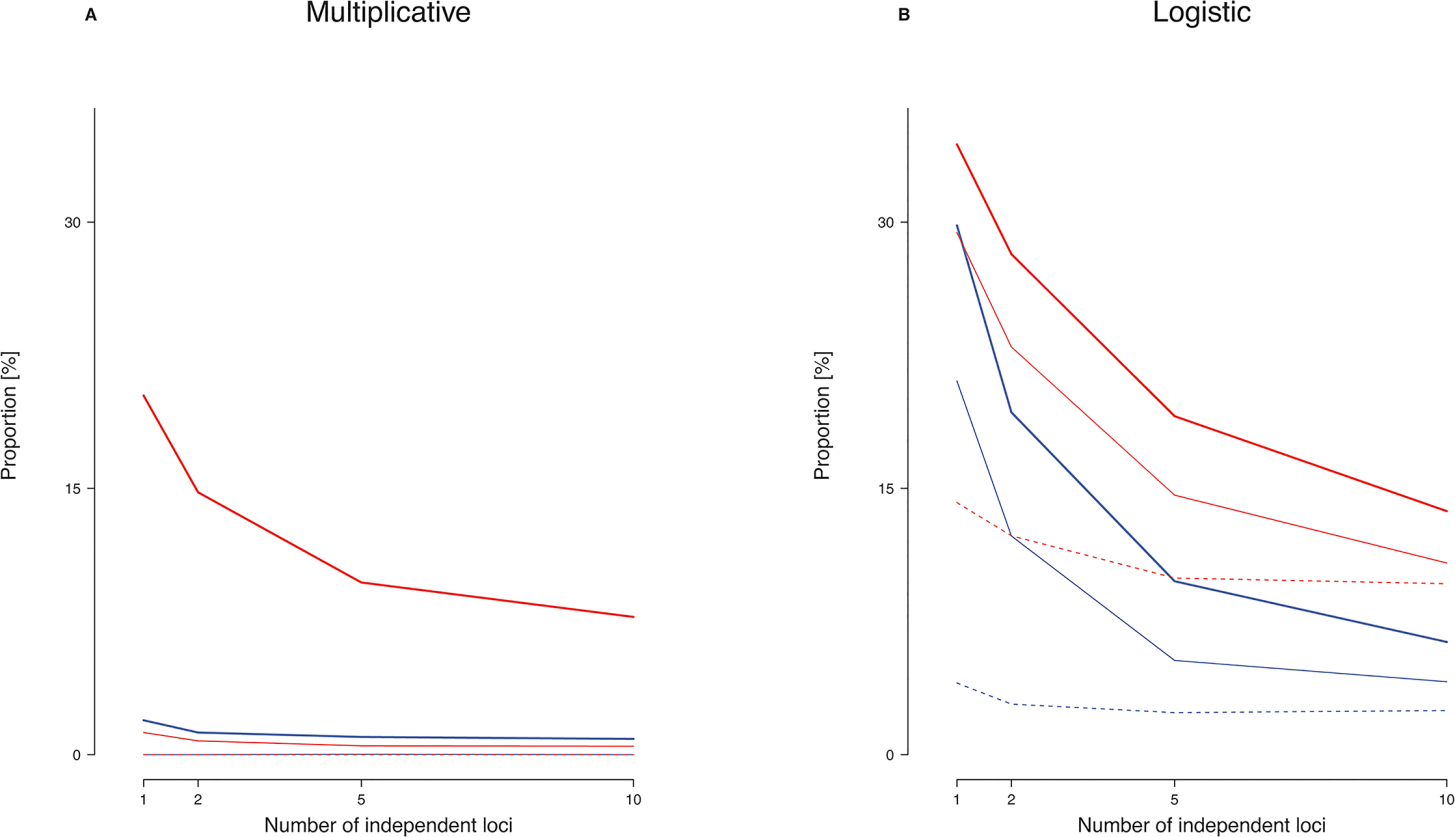
Observed proportion of epidemiologically protective causative mutations vs. number of unlinked loci underlying disease etiology. Bold solid line: pandemic disease (prevalence 10%-20%); Thin solid line: common disease (1%-5%); Dashed line: rare disease (0.1%-1.0%). (A) Multiplicative model with parameter value γ = 0.3 (blue) or γ = 0.1 (red). (B) Logistic model with parameter values *α* = -5; *β* = 1 (blue) or *α* = -5; *β* = 0.5 (red).

Analyses of pairs of unlinked (i.e. freely recombining) loci yielded results similar to those of the single-locus analysis (Figs 5 and 6). In fact, the median number of mutations per population, per case and per control remained virtually unchanged under both penetrance models and in all three prevalence categories (Table 3). However, the proportion of mutations with OR<1 was substantially reduced, particularly in instances of high prevalence and MAF (Table 4). This notwithstanding, the simulations revealed that, even with two unlinked loci contributing to the etiology of a given pandemic disease, up to 20% of mutations of at least moderate epidemiological effect size (OR>1.5 or OR<1/1.5) may still appear “protective” (Table 4). Simulations involving 5 or 10 unlinked loci (S1, S2, S3, S4 Figs and S3, S4, S5, S6 Tables) yielded smaller albeit still substantial proportions of apparently protective mutations. Simultaneous consideration of larger numbers of loci unfortunately turned out to be computationally prohibitive. However, the emerging trend (Fig 7) indicates that even polygenic diseases may feature a non-negligible proportion of causative mutations that appear protective in an epidemiological sense.

**Table 3:** Spectrum of causative mutations at two unlinked loci under two different penetrance models.

Model parameters	Median number of mutations (IQR)		
	Per population	Per case	Per control
**Rare disease (prevalence: 0.1–1%)**			
**Multiplicative**			
γ = 0.3	3 (2–5)	1.000 (1.000–1.000)	0.012 (0.007–0.019)
γ = 0.1	5 (3–7)	1.000 (1.000–1.029)	0.055 (0.029–0.077)
**Logistic**			
*α* = -5; *β* = 1	5 (3–8)	0.384 (0.200–0.553)	0.172 (0.080–0.264)
*α* = -5; *β* = 0.5	9 (5–15)	0.854 (0.614–1.052)	0.616 (0.422–0.781)
**Common disease (prevalence: 1–5%)**			
**Multiplicative**			
γ = 0.3	5 (3–8)	1.018 (1.002–1.051)	0.079 (0.051–0.105)
γ = 0.1	8 (5–13)	1.130 (1.036–1.284)	0.364 (0.251–0.436)
**Logistic**			
*α* = -5; *β* = 1	18 (13–25)	2.270 (2.040–2.480)	1.473 (1.268–1.645)
*α* = -5; *β* = 0.5	40 (29–54)	4.383 (4.145–4.609)	3.500 (3.253–3.709)
**Pandemic disease (prevalence: 10–20%)**			
**Multiplicative**			
γ = 0.3	10 (6–15)	1.240 (1.095–1.471)	0.453 (0.371–0.501)
γ = 0.1	22 (15–31)	2.426 (2.183–2.700)	1.879 (1.699–1.991)
**Logistic**			
*α* = -5; *β* = 1	35 (26–46)	4.012 (3.772–4.311)	2.784 (2.547–3.007)
*α* = -5; *β* = 0.5	72 (56–93)	7.704 (7.424–8.036)	6.319 (6.002–6.567)

IQR: inter-quartile range

**Table 4:** Proportion of epidemiologically protective mutations at two unlinked loci under two different penetrance models.

Model parameters	Percentage mutations with OR<1.0	Percentage mutations with OR>*m* or OR<1/*m* (percentage of these with OR<1)		Percentage mutations with OR<1.0 among those with frequency <*f*		
		*m* = 1.5	*m* = 2.0	*f* = 0.001	*f* = 0.01	*f* = 0.05
**Rare disease (prevalence: 0.1–1%)**						
**Multiplicative**						
γ = 0.3	0.00	100.0 (0.00)	100.0 (0.00)	0.00	0.00	0.00
γ = 0.1	0.00	100.0 (0.00)	100.00 (0.00)	0.00	0.00	0.00
**Logistic**						
*α* = -5; *β* = 1	2.85	90.54 (0.81)	78.51 (0.39)	0.00	0.00	3.78
*α* = -5; *β* = 0.5	12.33	68.97 (5.27)	44.17 (2.80)	0.00	0.00	14.02
**Common disease (prevalence: 1–5%)**						
**Multiplicative**						
γ = 0.3	0.00	99.96 (0.00)	99.86 (0.00)	0.00	0.00	0.00
γ = 0.1	0.78	97.35 (0.17)	93.76 (0.06)	0.00	1.57	1.07
**Logistic**						
*α* = -5; *β* = 1	12.32	79.14 (6.47)	63.24 (3.98)	0.00	9.09	10.59
*α* = -5; *β* = 0.5	22.97	64.70 (14.50)	42.99 (10.67)	0.00	17.66	21.36
**Pandemic disease (prevalence: 10–20%)**						
**Multiplicative**						
γ = 0.3	1.25	96.38 (0.40)	90.48 (0.17)	3.84	2.44	1.71
γ = 0.1	14.78	64.35 (7.61)	39.38 (4.95)	11.88	17.86	15.58
**Logistic**						
*α* = -5; *β* = 1	19.29	75.66 (13.32)	59.01 (10.09)	8.42	16.52	17.25
*α* = -5; *β* = 0.5	28.20	64.82 (21.39)	44.73 (17.55)	13.09	25.39	26.41

OR: odds ratio

**Fig 8:**
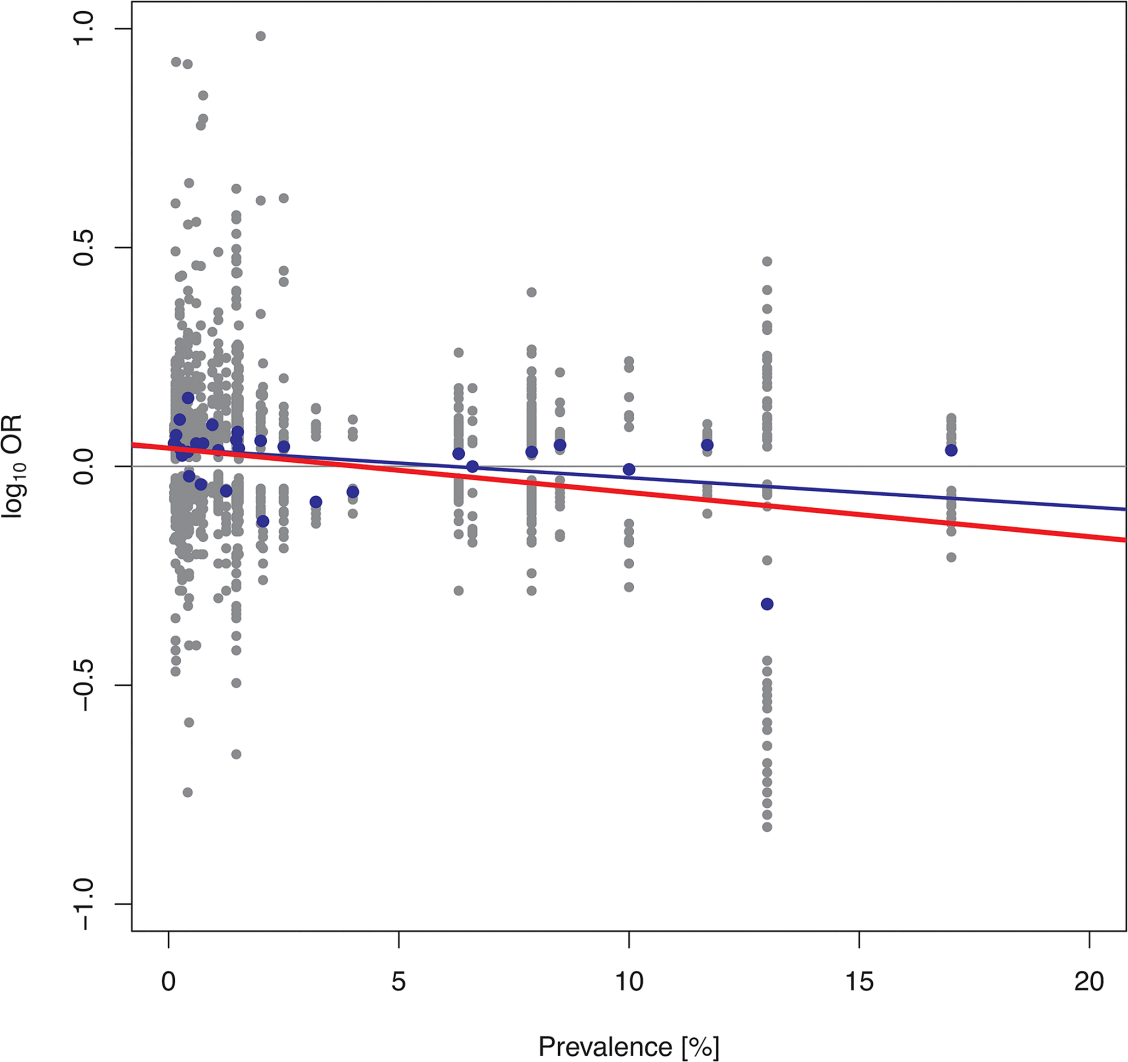
Effect size (log_10_OR) of trait-associated genetic variants vs. trait prevalence. The analysis was based upon data for 31 traits from the GWAS catalogue. Grey dots: trait-specific effect size (log_10_OR) in the GWAS catalogue with reported prevalence between 0.1 and 20% (i.e. 1834 associations with p<5x10^-4^); blue dots: median log_10_OR per trait; red line: linear regression line for log_10_OR versus trait prevalence (p<10^−15^); blue line: linear regression line for median log_10_OR versus trait prevalence (p = 0.049).

One predicted consequence of our findings would be that the effect size of disease-associated genetic variants should be negatively correlated with the prevalence of the respective trait. For example, simulation with a single causative locus and a logistic penetrance model (*α* = -5; *β* = 0.5) yielded median ORs (median log_10_ORs) of 1.84 (0.26), 1.61 (0.21) and 1.50 (0.18) for a rare, common and pandemic diseases, respectively (Fig 4D–4F). To test this prediction empirically, traits with at least 10 published genotype-phenotype associations at p<5×10^−8^ listed in the GWAS catalogue [30] were scrutinized for variants that had both an OR and the MAF reported (S2 File). When we focused upon those 31 traits with reliable prevalence information (range: 0.1% to 20%), a total of 1834 associations with p<5×10^−4^ became available for analysis (S2 Table). These data indeed revealed a statistically significant trend towards smaller ORs with increasing prevalence (p<10^−15^ for single-variant ORs and p = 0.049 for phenotype-wise median ORs, respectively; Fig 8). This observation was robust in the sense that it did not depend upon a few particularly prevalent clinical phenotypes. Confining the analysis to associations of genome-wide significance (p<5×10^−8^, 31 traits, 1298 associations) did not change this trend (p = 1.9×10^−6^ and p = 0.051, respectively), which also remained when traits with prevalence <0.1% were included (43 traits, 2178 associations, p<10^−15^ and p = 0.01, respectively) or when the prevalence was confined to values <15% (30 traits, 1809 associations, p<10^−15^ and p = 0.01, respectively). Finally, confining the analysis to those 1418 SNPs associated with a trait with a prevalence ranging from 0.1% to 20% and where the derived allele could be identified unambiguously (S2 Table) also revealed a trend towards lower ORs with increasing prevalence even although this result failed to attain statistical significance (p = 0.38 and p = 0.71, respectively; S5 Fig).

## Discussion

In those genetic epidemiological studies that are based upon statistical genotype-phenotype relationships alone, drawing causal inference from ORs can be problematic for common and pandemic complex diseases for which the presence of a particular mutation is rarely necessary (let alone sufficient). Indeed, our simulation-based analyses revealed that causative inherited mutations may well be more frequent in the clinically unaffected than in the affected portion of a population, thereby rendering them “protective” rather than predisposing for these diseases in an epidemiological sense. This counterintuitive phenomenon results from confounding of the disease association of one mutation by the absence of other mutations necessary for the former to exert its effect in a given individual.

A negative correlation between causative mutations can potentially arise due to the tree structure of the human gene genealogy. More specifically, causative mutations that occur early on in the history of a given population will be wide-spread but still have to accumulate other, mostly younger mutations on the same haplotype for disease to occur in the current generation. Younger mutations, on the other hand, are confined to different sub-trees so that their co-occurrence with one another is less likely at the population level. As a net result, some mutations, both old and young, may end up at the tips of sub-trees poor in complementing (younger) mutations. Under the penetrance models employed here, such a negative correlation is more likely to become evident for common and pandemic diseases than for rare diseases because the former will inevitably have the more comprehensive mutational spectrum. Indeed, notwithstanding alternative explanations, our empirical observation of a negative correlation between ORs and disease prevalence in published GWAS reports lends support to the validity of this view because such a correlation would be predicted by the above mechanism of confounding.

Since the described phenomenon of effect size obfuscation is due to a negative correlation between causative mutations, it may be surmised that oligogenic and polygenic diseases would be less likely to be affected, given the likelihood of recombination eradicating the necessary correlations with time. However, our simulations of diseases etiologies involving up to 10 unlinked loci indicate that this decay with gene number of the implied inter-mutation correlation may be smaller than expected, which means that our findings are relevant to monogenic and oligogenic diseases alike and may, to some extent, even apply to polygenic diseases.

At first glance, the confounding of genotype-phenotype relationships by the underlying gene genealogy may appear reminiscent of other, similarly counterintuitive phenomena. For example, ‘synthetic association’ [13–16] conceptualizes the notion that a common non-causative genetic variant may become disease-associated due to the chance accumulation of rare causative variants *in cis*. By contrast, the confounding mechanism described herein refers exclusively to truly causative mutations and addresses their genealogy-actuated relationship. Moreover, studies of synthetic association [13] so far have paid little attention to the effect sizes of the rare (causative) variants but have instead focused mainly upon the common (non-causative) variant. ‘Flip-flop’ association [12] is another enigma that signifies a frequent reverting role of the risk allele in different studies of one and the same genotype-phenotype relationship. Flip-flop associations are potentially explicable by chance differences in linkage disequilibrium between different samples from the same population. Here, we repeatedly simulated a whole population so that the ensuing disease prevalence, allele frequencies and potentially misleading OR were true (population-wide) values, not estimates subject to sampling error. Confounding by gene genealogy is also different from ‘indirect association’ [17] although the core ideas of the two concepts partly overlap. Nevertheless, while indirect association focuses upon the consequences of linkage disequilibrium and gene-gene interaction between a non-causative and a causative variant, our analysis was concerned exclusively with causative mutations that become subject to confounding themselves.

Our results imply that the functional follow-up of disease-associated genetic variants could in some cases be misguided with the consequence that truly causative mutations might inadvertently and inappropriately be excluded from further investigation. While the protective effect of a causative mutation may pose minor problems in terms of its initial statistical evaluation, an OR<1 would be difficult to reconcile with the damaging character of the mutation in subsequent bioinformatics analyses. Since expensive functional studies *in vitro* or *in vivo* usually require solid theoretical evidence for causality, such inconclusiveness is likely to be a major deterrent to further research on the variant in question. A genealogy-actuated negative correlation between causative mutations may also explain why many genotype associations with complex diseases have often been difficult to replicate, because the identity, frequency and degree of association of the negatively correlated causative mutations are likely to differ between populations. Consistent with this postulate, several disease associations reported in the literature have been with different variants in the respective discovery and replication analyses [32–36].

Our results suggest that many causative mutations are unlikely to be identified by association analysis based upon case-control comparisons, thereby rendering questionable the frequent claim that the success prospects of GWAS depend heavily upon sample size [2]. Moreover, great store has been set by employing next-generation sequencing (NGS) to identify rare, and therefore potentially younger, causative mutations. Some current strategies for the statistical analysis of NGS data aim to detect genes that are enriched in mutations in cases as compared to controls [37–43]. As has been shown here, such a strategy could be seriously compromised, particularly for common and pandemic diseases, if a certain threshold number of mutations were to be required for the functionality of a relevant gene to be sufficiently impaired to yield a clinical phenotype [19]. One possible way out of this dilemma could be to incorporate population genealogy more explicitly into the statistical evaluation of NGS data, either analytically or by way of simulation. Indeed, the development of the necessary methodology may well constitute an interesting new field of future genetic epidemiological research. Furthermore, one way to identify candidate variants for a genealogy-actuated negative correlation would be to search for apparently protective variants that have been subject to negative selection or conversely, apparent risk variants that have been subject to positive selection. Finally, it is worthy of note that linkage-based genetic epidemiological studies would not be affected by the confounding phenomenon described here because they exploit the co-segregation of genotypes and phenotypes in families rather than their co-occurrence in unrelated individuals.

Although the coalescent is the method of choice to model gene genealogies, the many assumptions made in coalescence theory have prompted some criticism of the approach [23]. In particular, the basic coalescent used in our study is valid only for an idealized population, i.e. one with non-overlapping generations, constant size, random mating and a lack of selection and recombination [22]. In reality, these assumptions are rarely if ever met. Thus, future studies of the confounding phenomenon described herein may well wish to adopt more complex genealogy models, although we expect our conclusions to remain largely unchanged. For example, the Moran model allows for overlapping generations although it can reasonably be approximated by the basic coalescent via an adjustment of the time scale [22]. Consideration of population growth would lead to shorter branches at the root and longer branches at the tips of the coalescence tree [22,44] but the possibility of a negative correlation between causative mutations would still remain. The same is true for population structure, which may even exacerbate the described phenomenon because population structure tends to promote the separation of younger mutations in a genealogical tree. We would also maintain that, in view of the multifactorial nature of many common diseases, selection of single causative mutations is likely to have been weak up to the point that some deleterious mutations could have become fixed in human evolution at a rate similar to that of neutral variation [45]. Therefore, selection may be neglected here, at least for common and pandemic diseases where the impact of a single causative mutation on biological fitness is expected to be minor. Finally, models taking recombination into account would allow the investigation of larger genes or gene regions. However, the simulations would become much more complicated because a recombination coalescent comprises random graphs instead of a random tree [23], and it is unclear whether this additional level of complexity would qualitatively change our conclusions.

The same effect size was assumed in our simulations for frequent and rare mutations. Although rare mutations with strong effects undoubtedly would be easier to detect through association studies (e.g. [46]), this does not preclude the possibility that in reality many rare variants also exert only modest effects. Consequently, assuming equal effect sizes for rare and frequent variants is not uncommon for simulation studies in genetic epidemiology (e.g. [26]). Furthermore, whilst the total population number of causative mutations in our study may appear somewhat high under some scenarios, these numbers nevertheless recall the shift in mutation-drift equilibrium towards an accumulation of rare variants in recent human history [47]. Indeed, substantial heterogeneity of causative alleles at a single disease locus is not uncommon even for monogenic subtypes of human diseases, including breast cancer due to *BRCA1* mutations [48,49].

Our consideration of pairs or groups of unlinked loci revealed that the proportion of apparently protective mutations arising along a given genealogy is only slightly smaller than for a single locus, which implies that recombination should only slightly affect the potential for confounding described herein. Moreover, although our simulations were confined to haploid loci, it is important to note that diploid inheritance is equally likely to suffer from the described decoupling of causality and the epidemiological effect size of individual mutations.

Finally, it could be argued that higher disease prevalence would automatically lead to larger samples so that variants with small effect size should not be missed particularly for common or pandemic diseases. The truth of this assertion notwithstanding, sample size seems to be of minor relevance in the present context because, even for a disease with 0.1% prevalence, a medium-sized population of 40 million (i.e. still smaller than that of the UK, France or Germany, which have been a strong focus of GWAS) would still contain at least 40,000 cases. Thus, prevalence is not a rate limiting factor for achieving the usual GWAS sample sizes of 3000 to 5000 cases.

In conclusion, genetic studies of common human diseases should take into account the possibility that some causative genetic variants could pose as protecting rather than predisposing in classical case-control comparisons. In cases of discordant epidemiological and bioinformatics evidence, both (or all alleles) of a significantly associated variant should be considered putatively causative in functional follow up analyses. Moreover, collapsing of rare variants from next-generation sequencing in case-control studies should be done with great care, acknowledging the fact that the opposing effects of truly causative mutations may cancel out. Finally, it may well be that we shall have to come to terms with the fact that some mutations causing complex human disease are inherently unidentifiable *ab initio* by case-control association studies.

## Supporting Information

S1 Fig Epidemiological effect size of causative mutations at 5 unlinked loci under two different penetrance models. See legend to Fig 3 for details.
(PDF)

S2 Fig Relationship between epidemiological effect size (log_10_OR) and frequency of causative mutations at 5 unlinked loci under two different penetrance models. See legend to Fig 4 for details.
(PDF)

S3 Fig Epidemiological effect size of causative mutations at 10 unlinked loci under two different penetrance models. See legend to Fig 3 for details.
(PDF)

S4 Fig Relationship between epidemiological effect size (log_10_OR) and frequency of causative mutations at 10 unlinked loci under two different penetrance models. See legend to Fig 4 for details.
(PDF)

S5 Fig Effect size (log_10_OR) of trait-associated genetic variants vs. trait prevalence. The analysis was based upon data for 31 traits taken from the GWAS catalogue. All ORs refer to the derived allele. Grey dots: trait-specific effect size (log_10_OR) in the GWAS catalogue for traits with reported prevalence between 0.1 and 20% (i.e. 1418 associations with p<5x10^-4^); blue dots: median log_10_OR per trait; red line: linear regression line for log_10_OR vs. trait prevalence (p = 0.38); blue line: linear regression line for median log_10_OR vs. trait prevalence (p = 0.71).
(PDF)

S1 File Source code of simulations performed. (TXT)

S2 File Extract from GWAS catalogue used for the analysis. (TXT)

S1 Table Synonymous trait names for reports of genome-wide significance identified from the GWAS catalogue. (PDF)

S2 Table List of SNP associations of genome-wide significance as identified from the GWAS catalogue and considered in this study. (PDF)

S3 Table Joint mutational spectrum at 5 unlinked loci under two different penetrance models. (PDF)

S4 Table Proportion of epidemiologically protective mutations at 5 unlinked loci under two different penetrance models. (PDF)

S5 Table Joint mutational spectrum at 10 unlinked loci under two different penetrance models. (PDF)

S6 Table Proportion of epidemiologically protective mutations at 10 unlinked loci under two different penetrance models. (PDF)
