# Supplementary figures and images for: Mutations Causing Complex Disease May under Certain Circumstances Be Protective in an Epidemiological Sense
Source: PLoS One. 2015 Jul 10;10(7):e0132150. doi: 10.1371/journal.pone.0132150 (PMC4498598; doi:10.1371/journal.pone.0132150)

Rare Disease

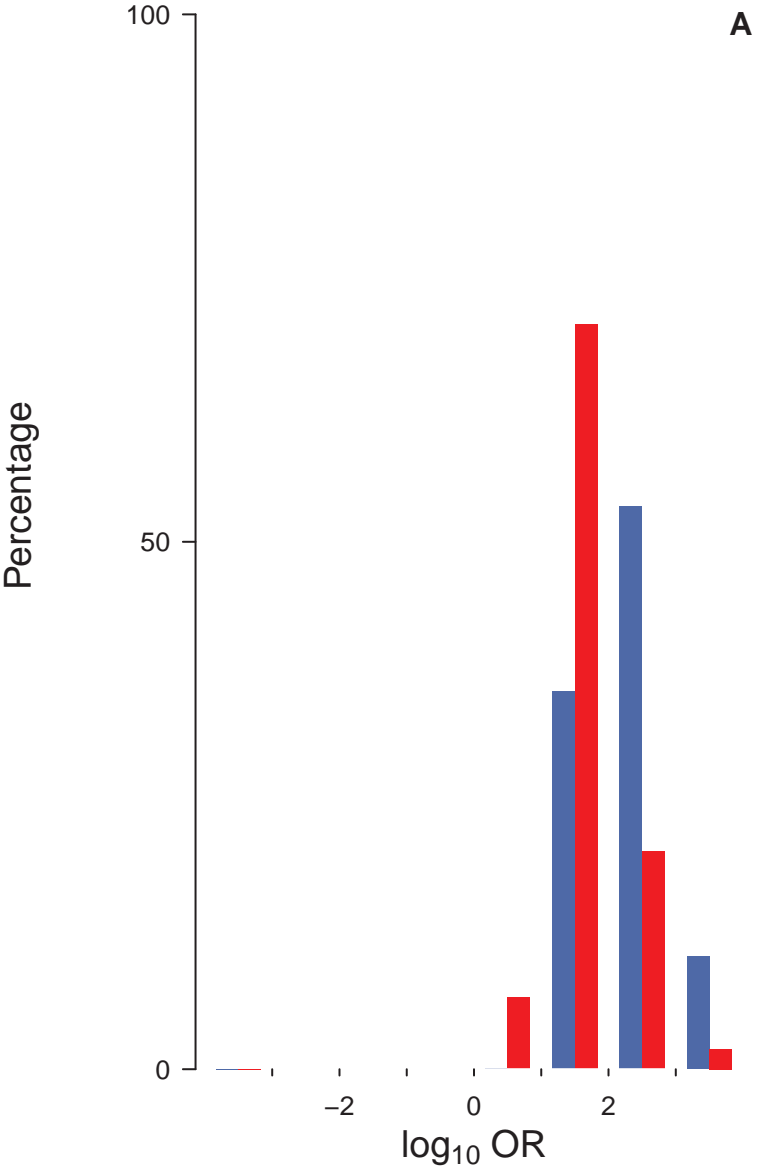

Common Disease

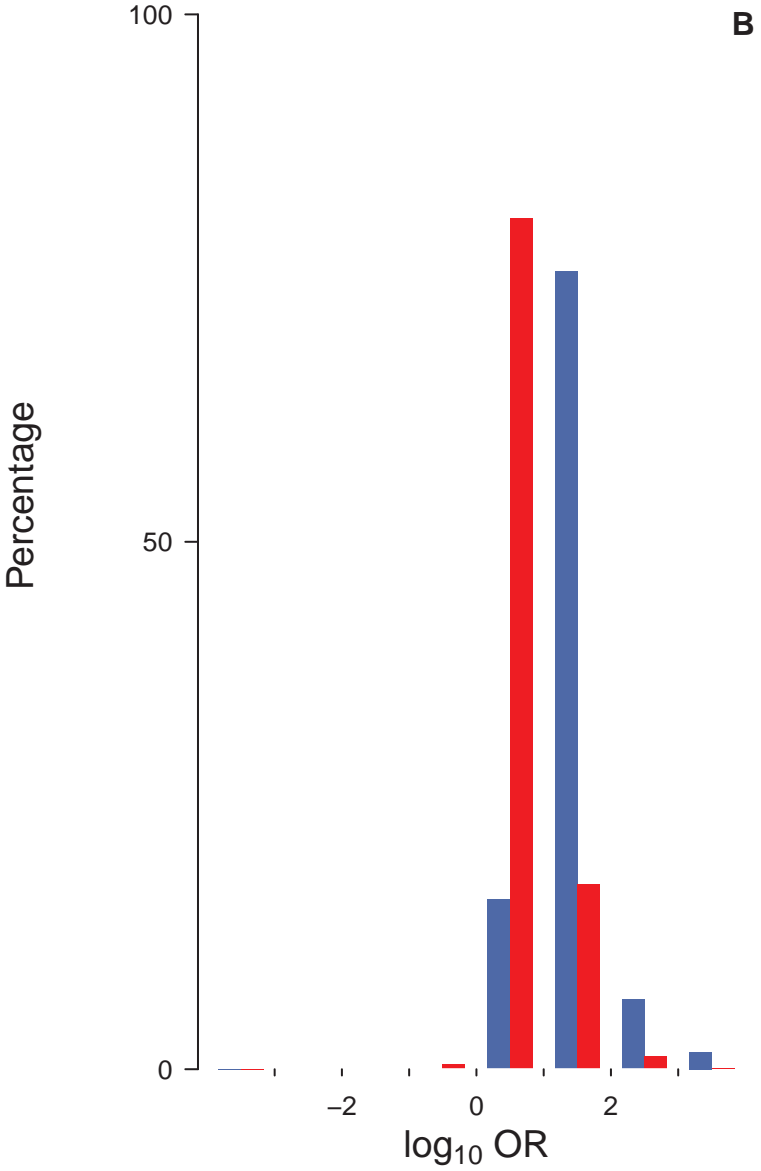

Pandemic Disease

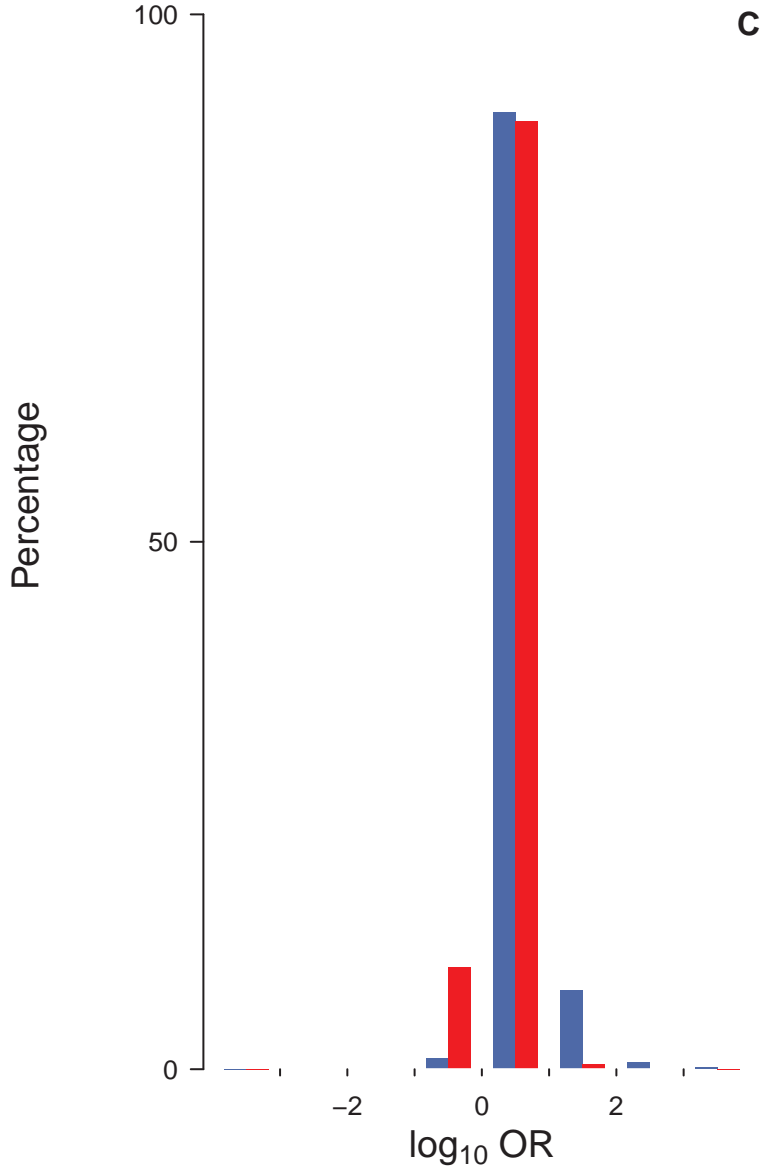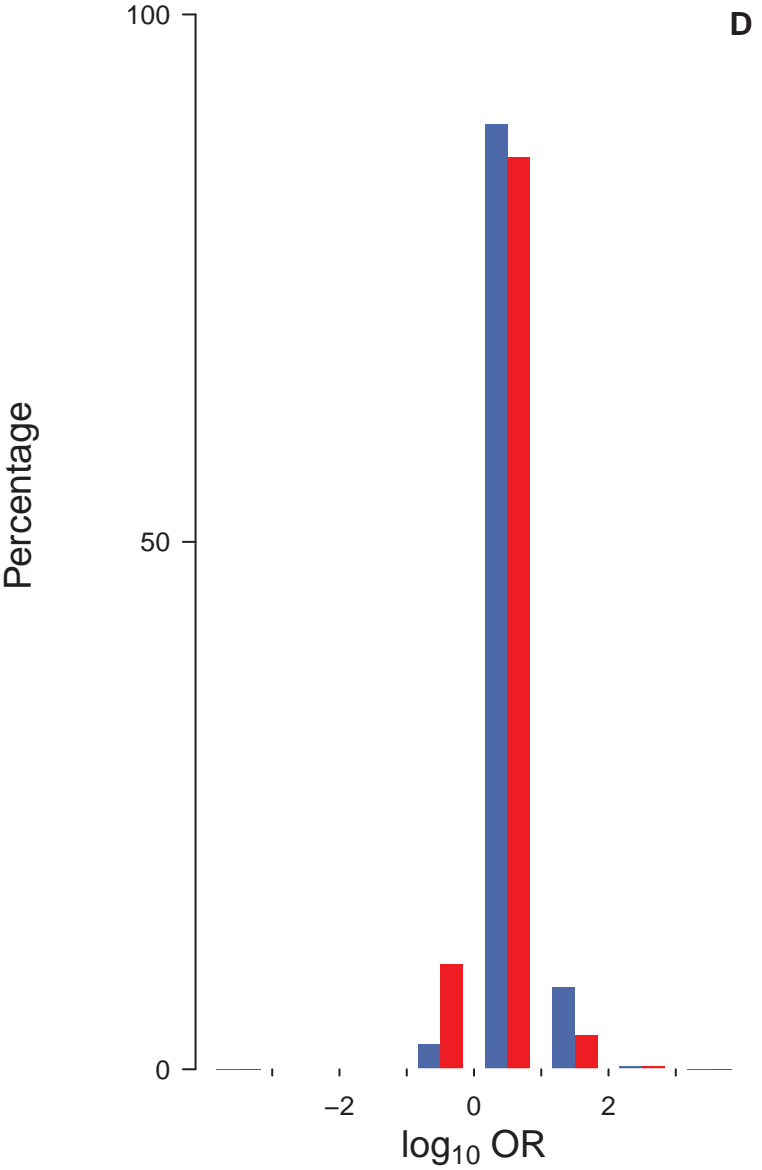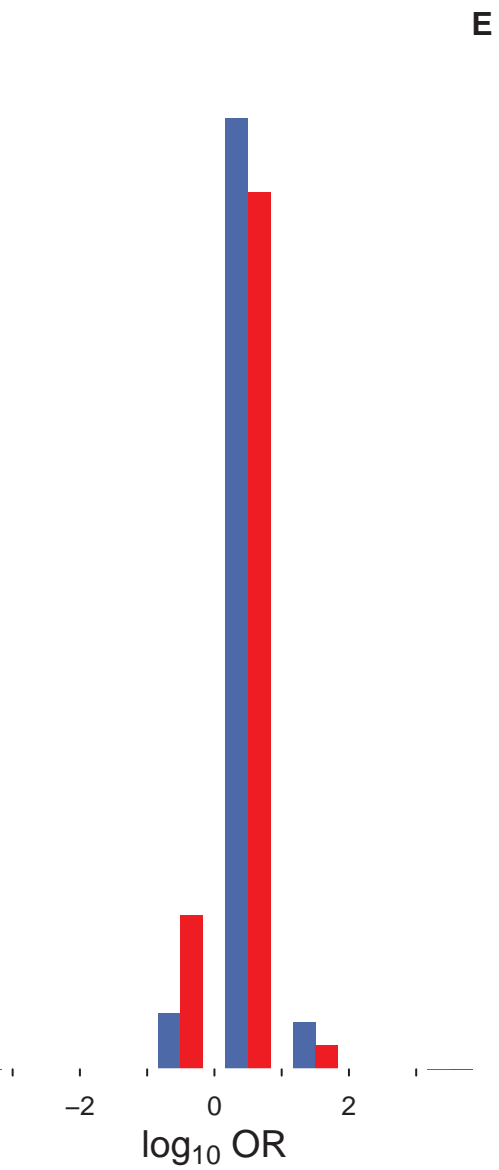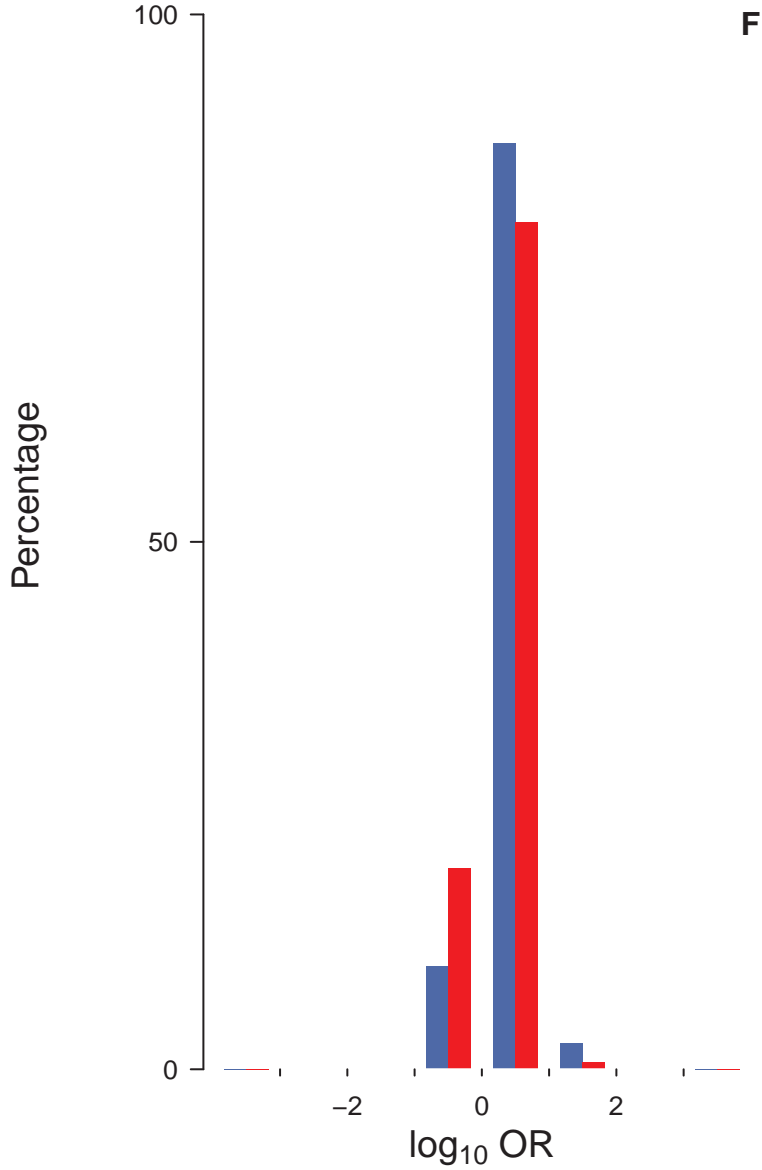

Supplement: S1 Fig — See legend to Fig 3 for details. (PDF) [file pone.0132150.s001.pdf]

Rare Disease

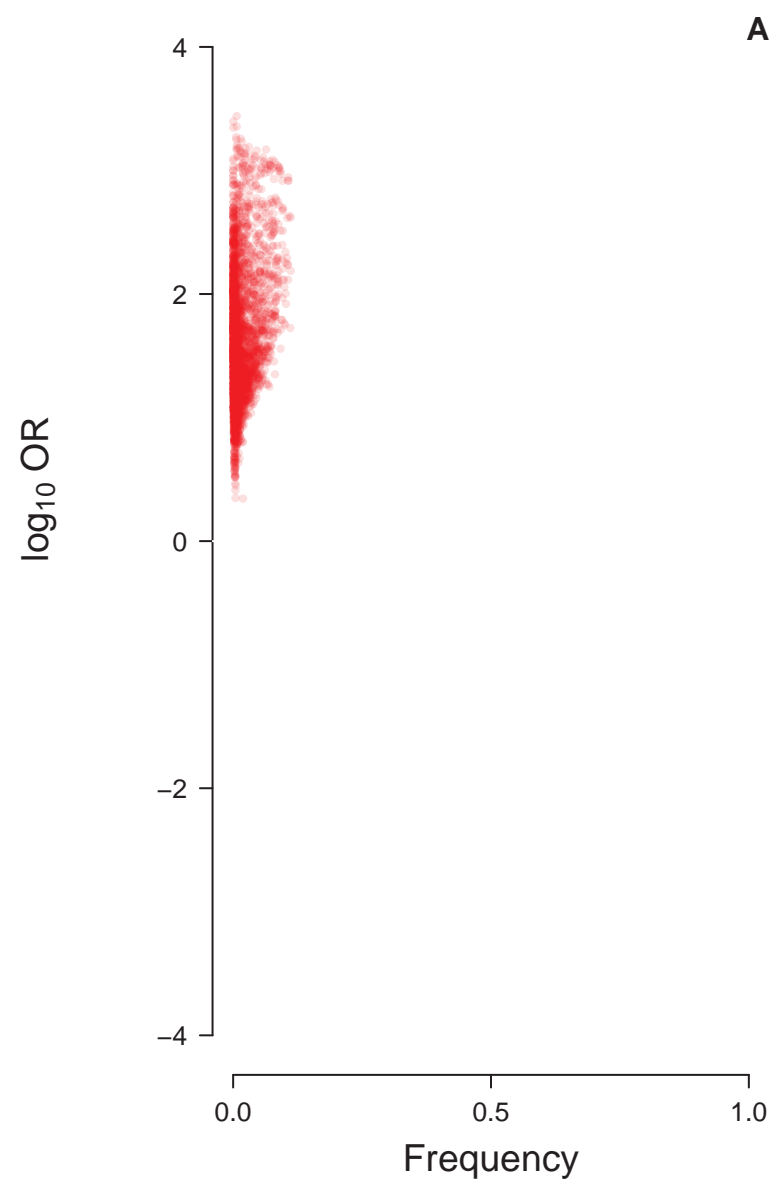

Common Disease

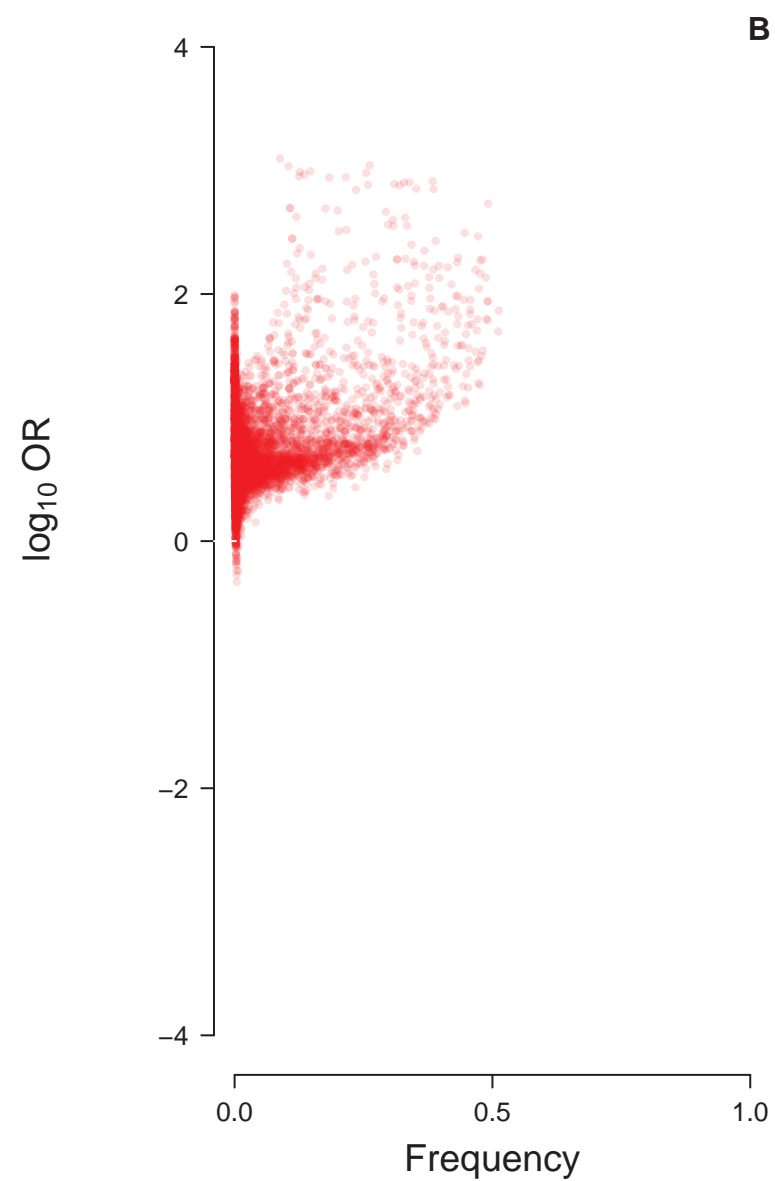

Pandemic Disease

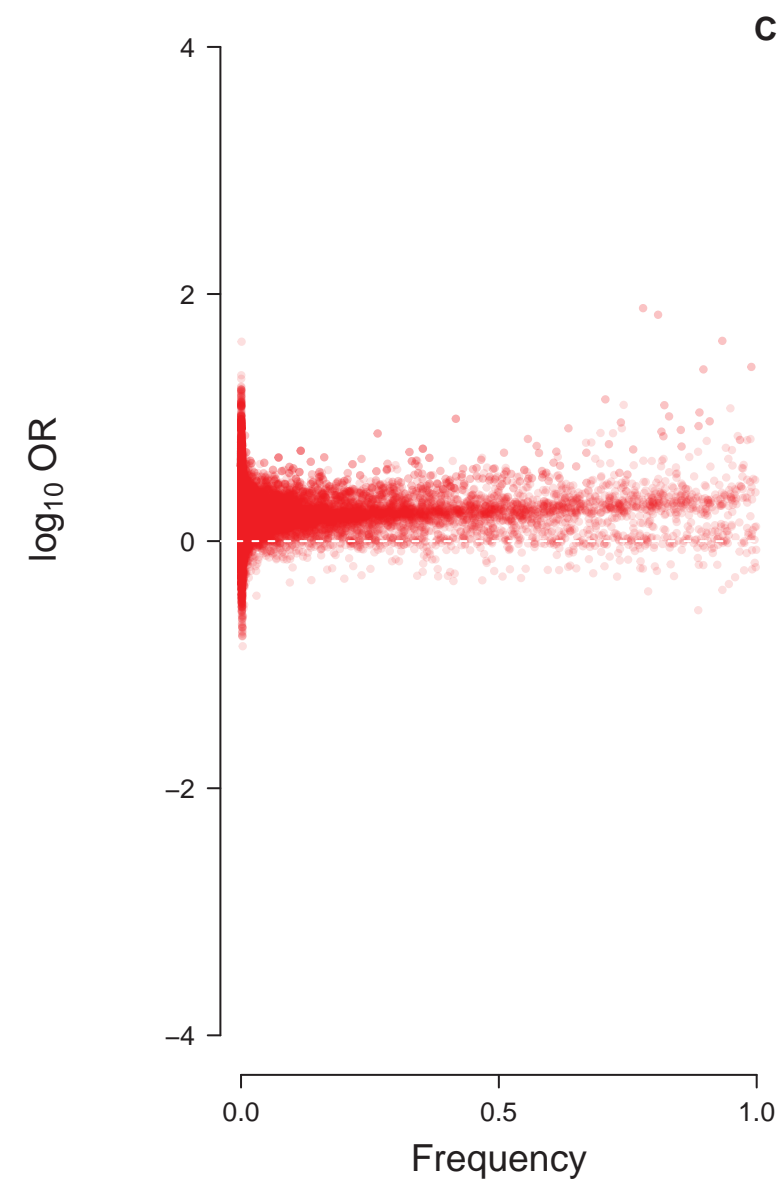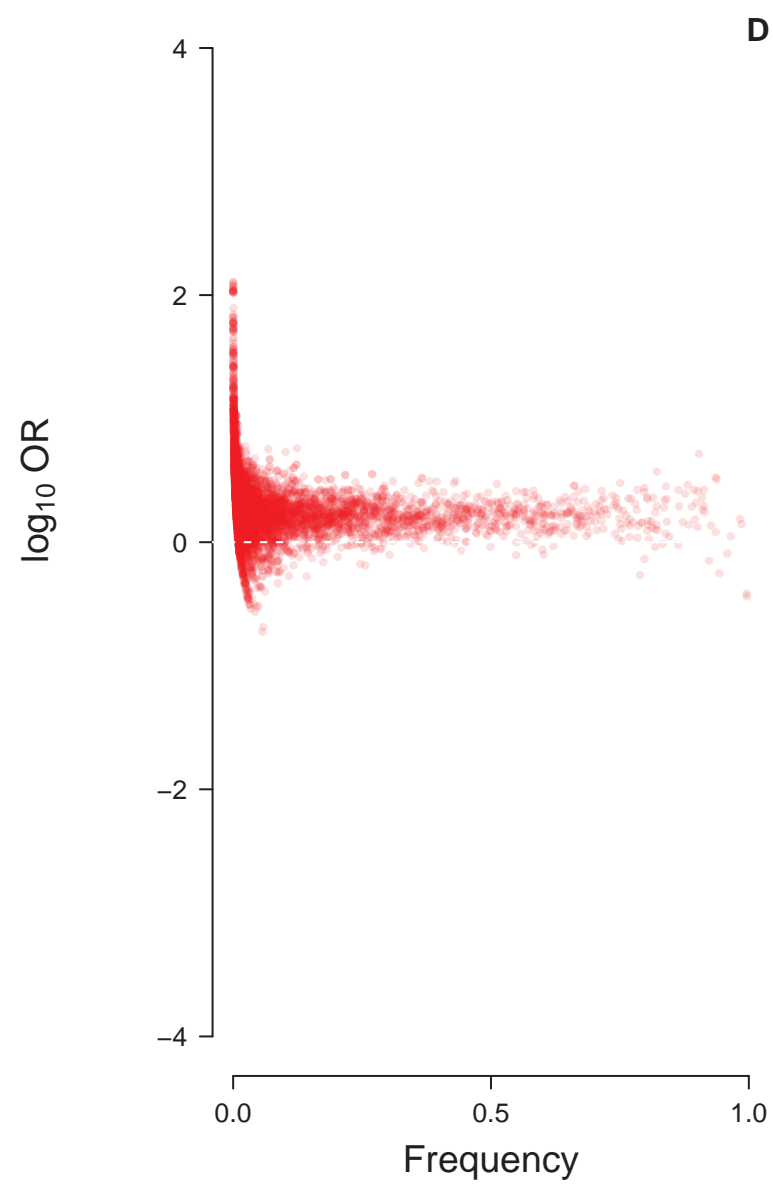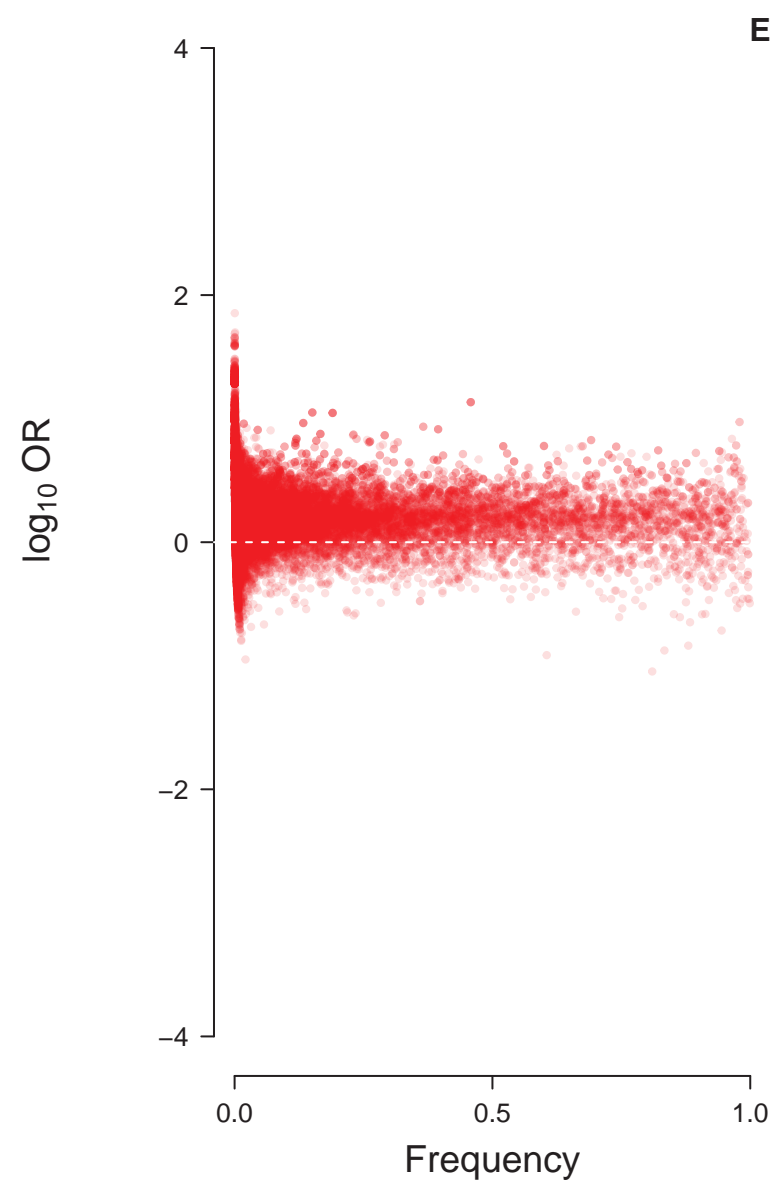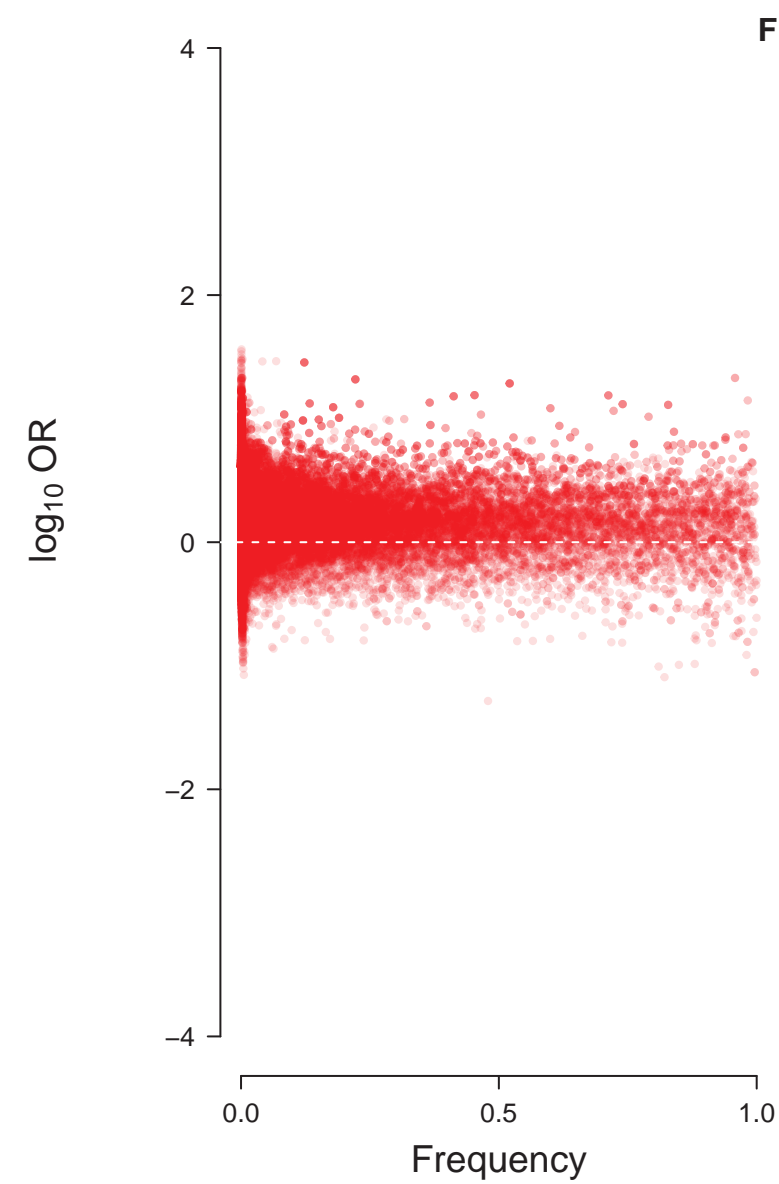

Supplement: S2 Fig — See legend to Fig 4 for details. (PDF) [file pone.0132150.s002.pdf]

Rare Disease

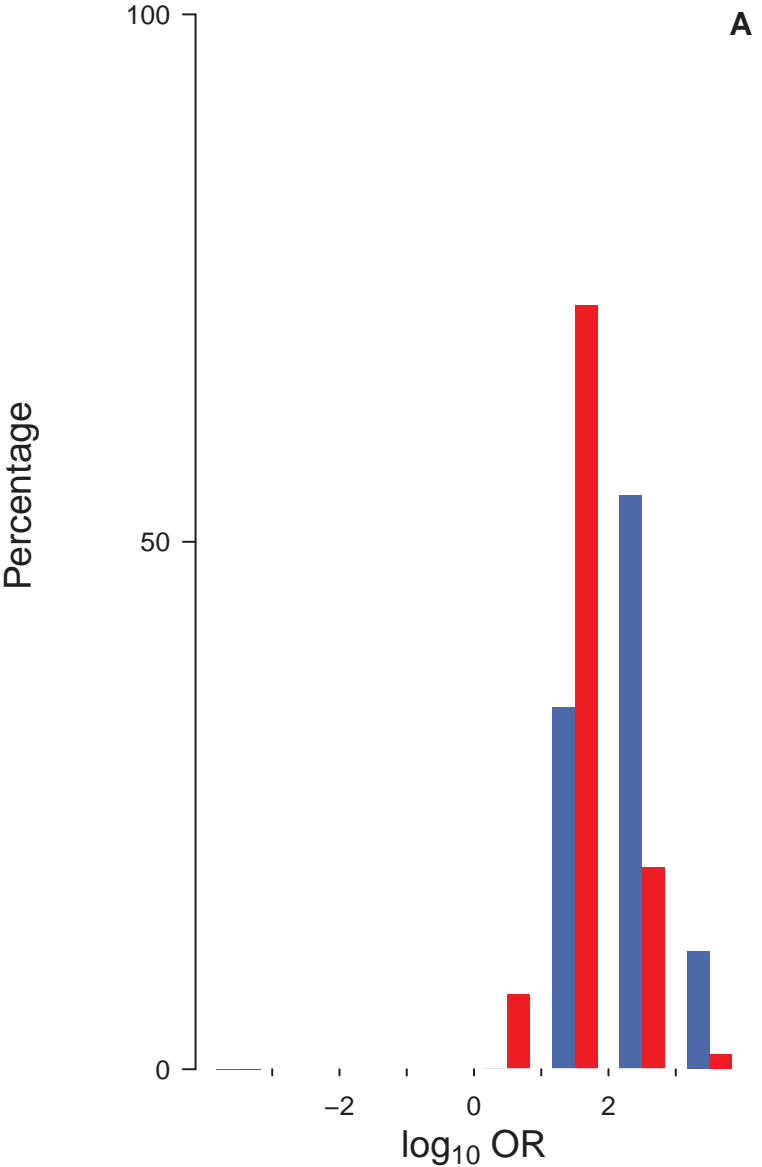

Common Disease

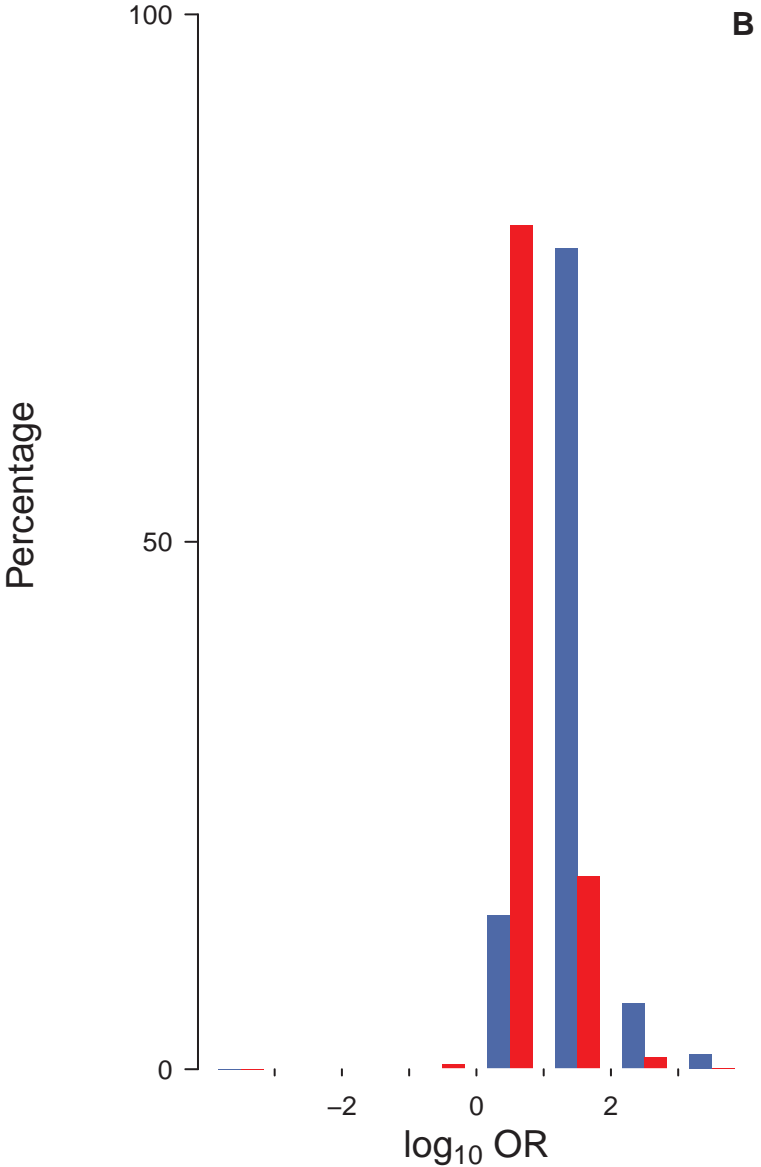

Pandemic Disease

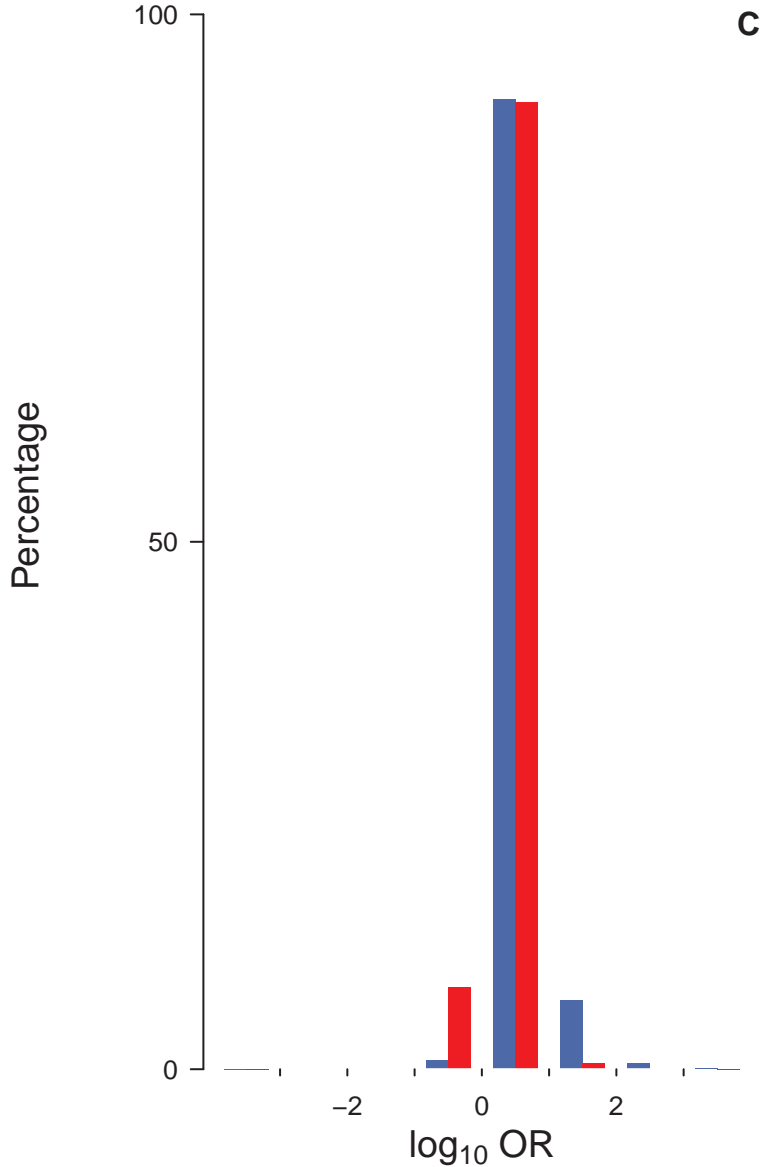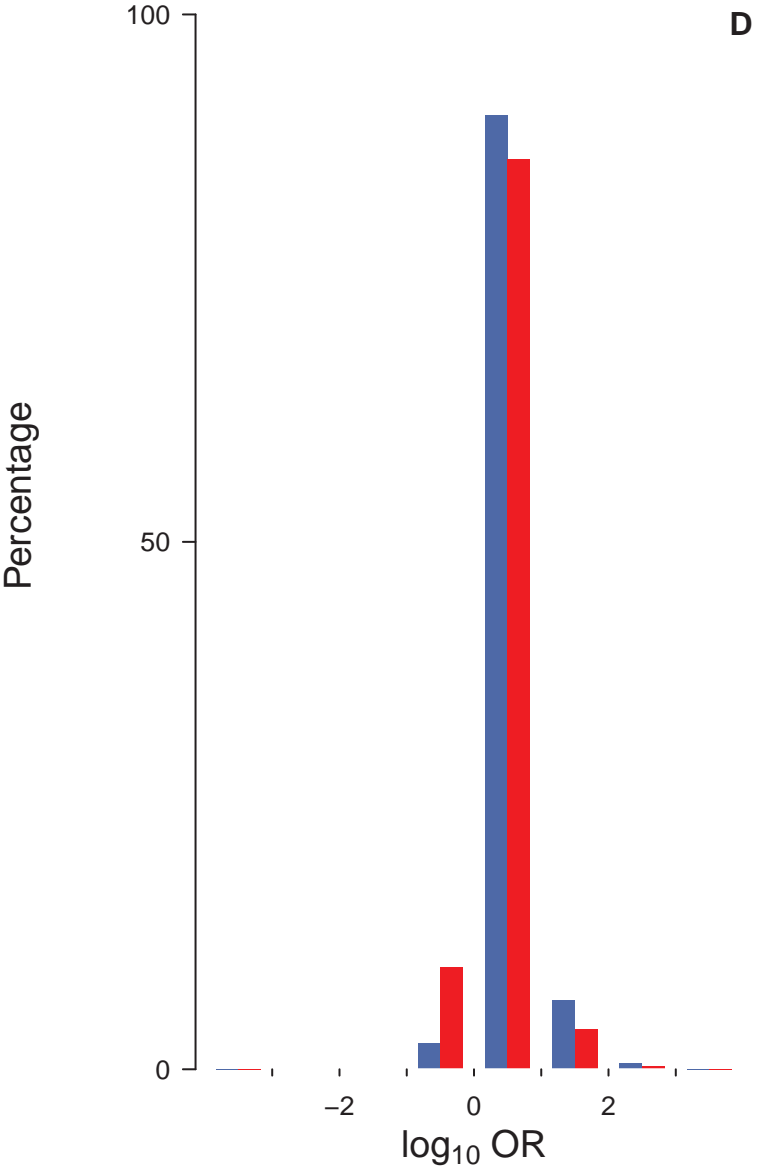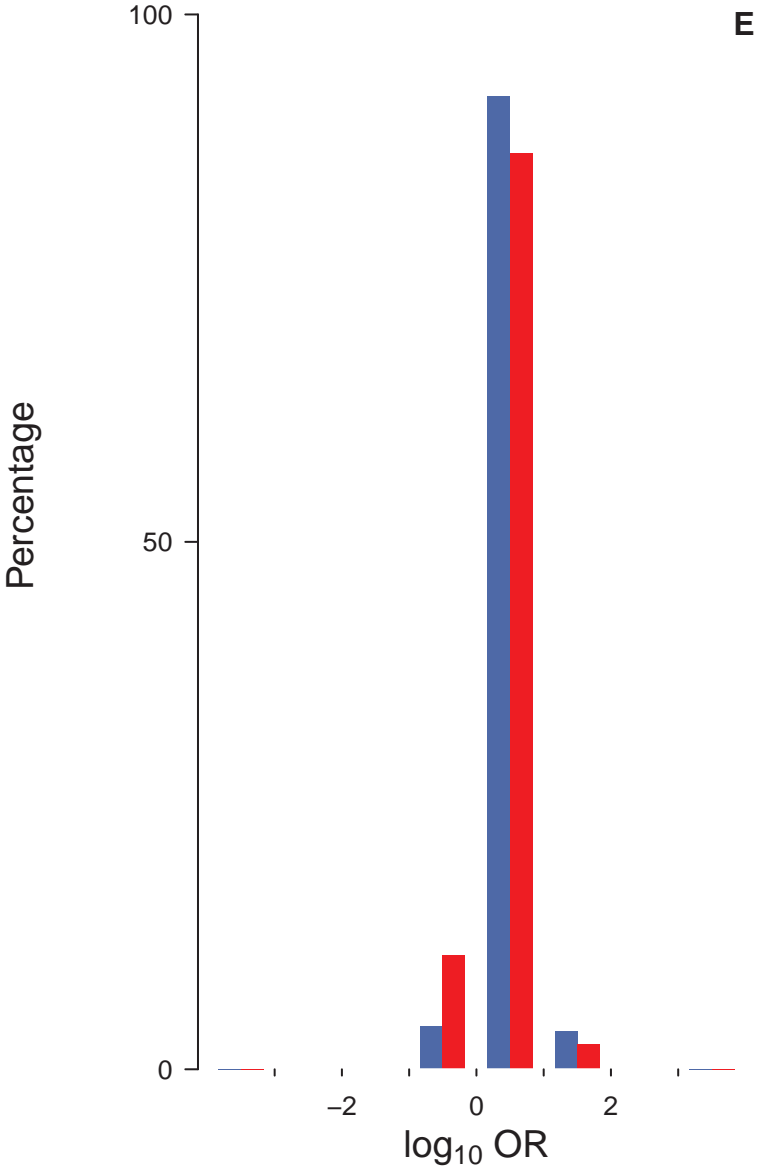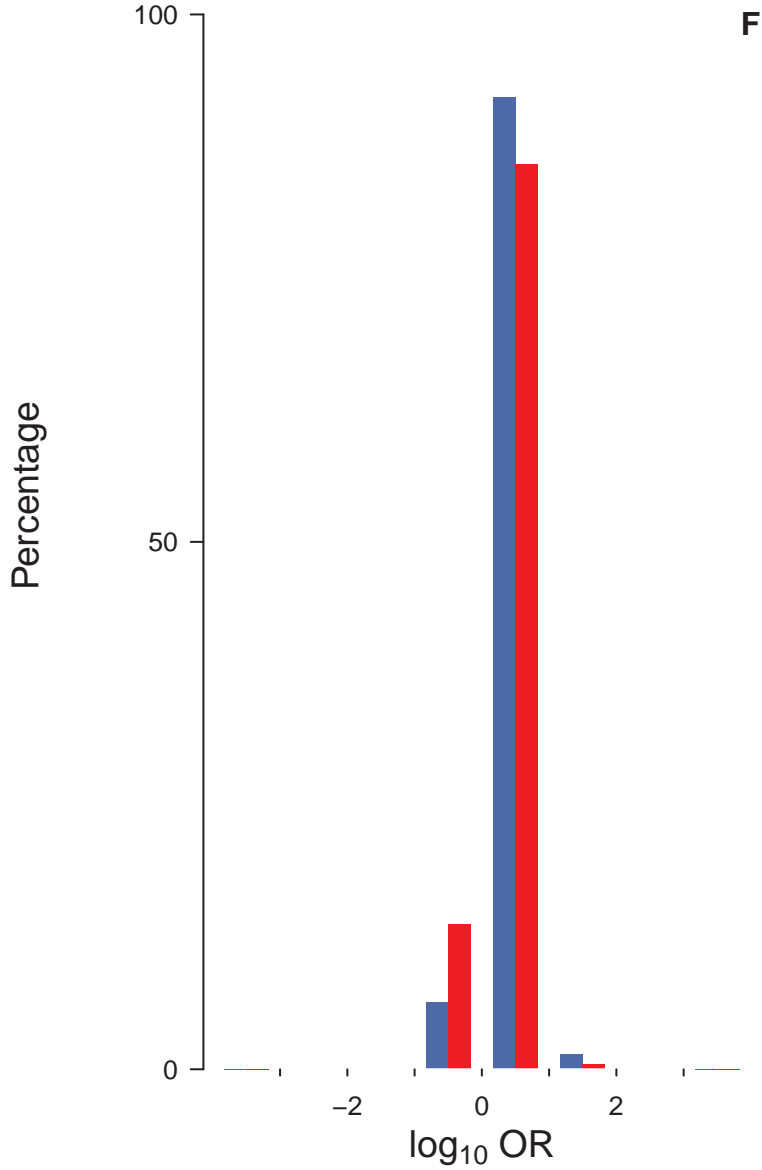

Supplement: S3 Fig — See legend to Fig 3 for details. (PDF) [file pone.0132150.s003.pdf]

Rare Disease

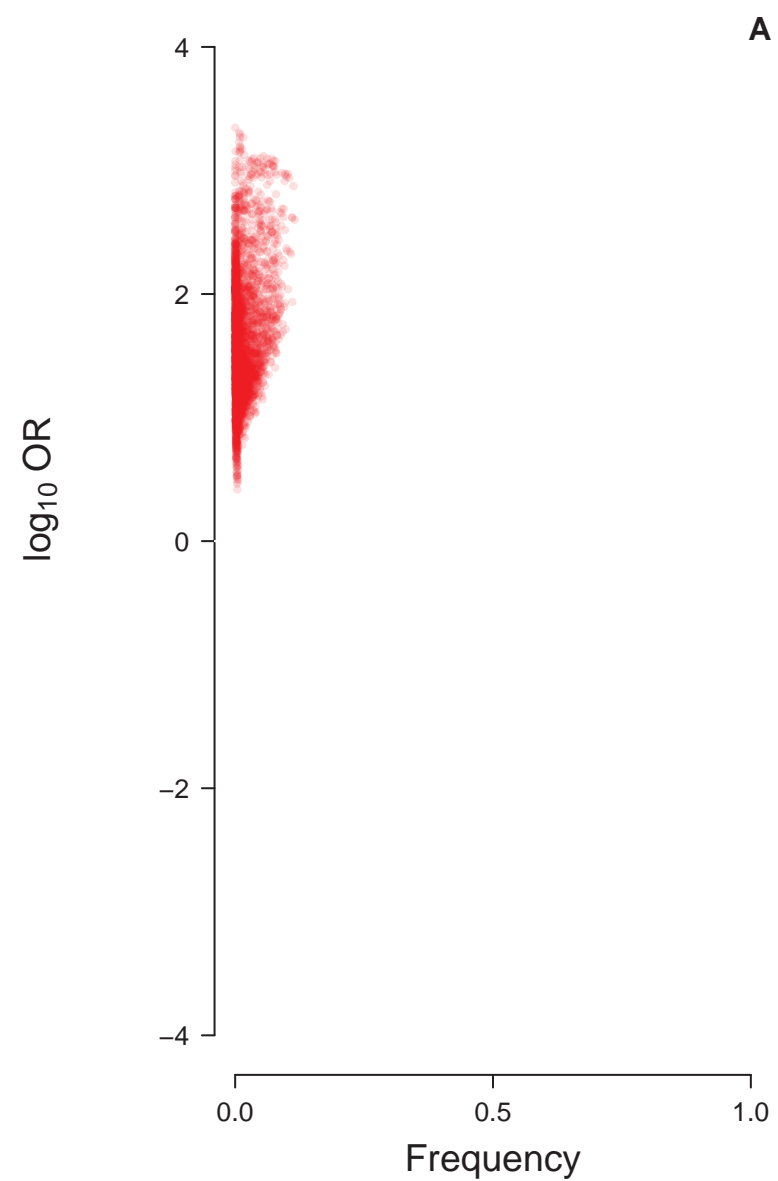

Common Disease

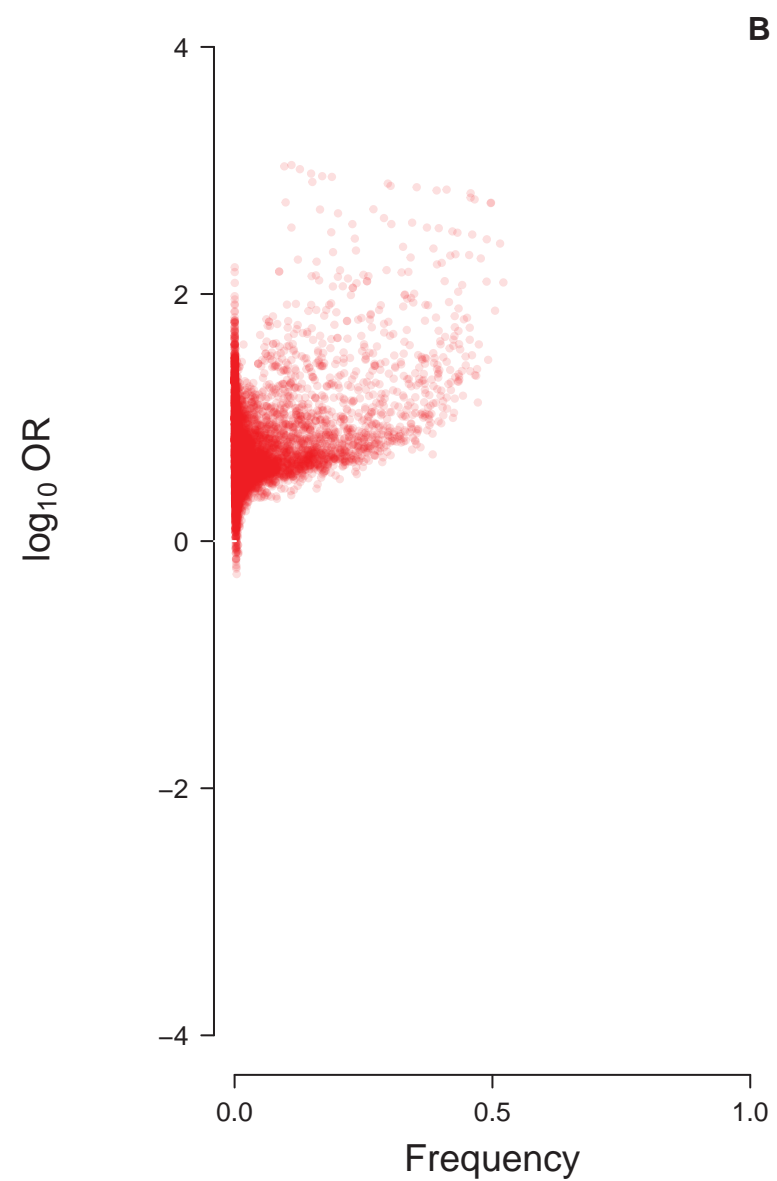

Pandemic Disease

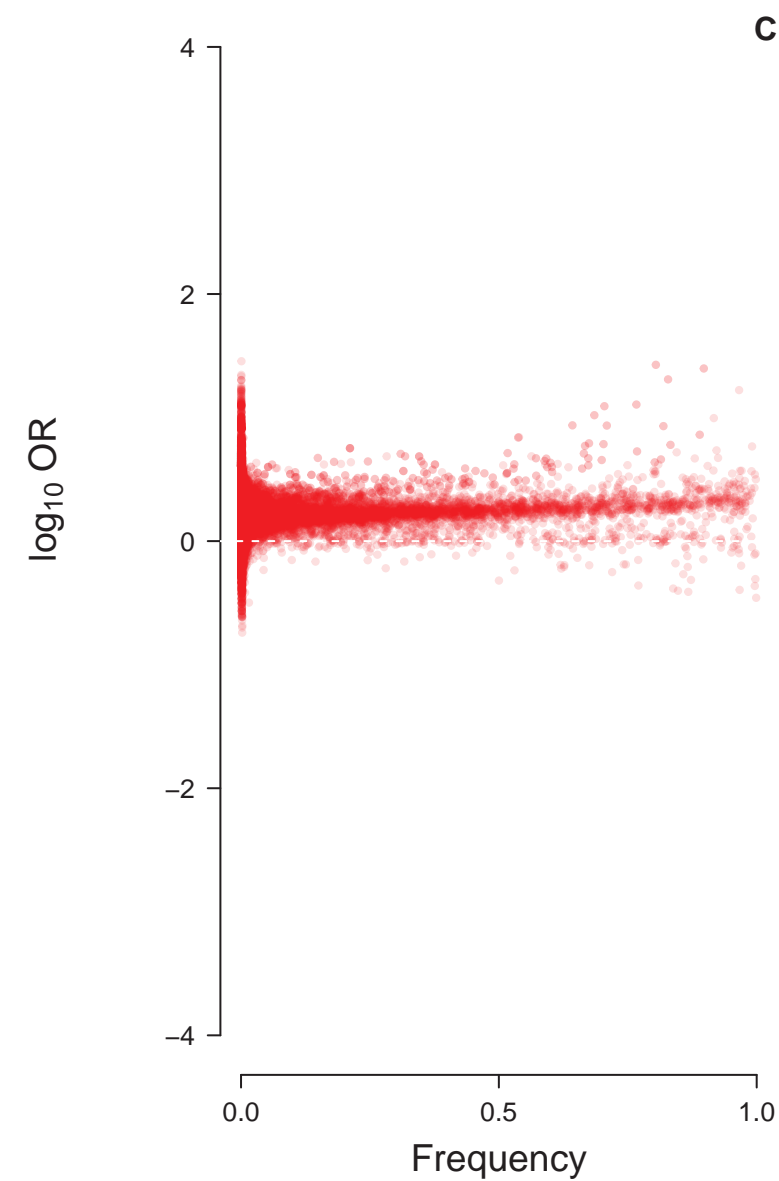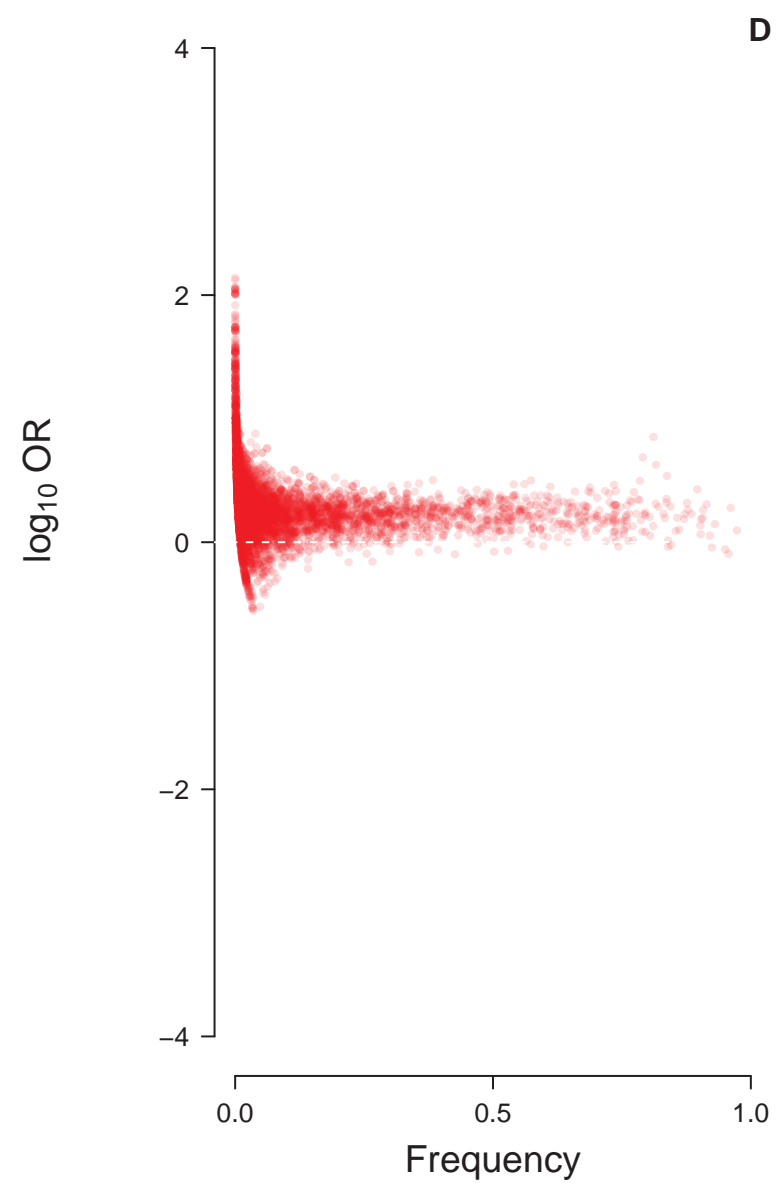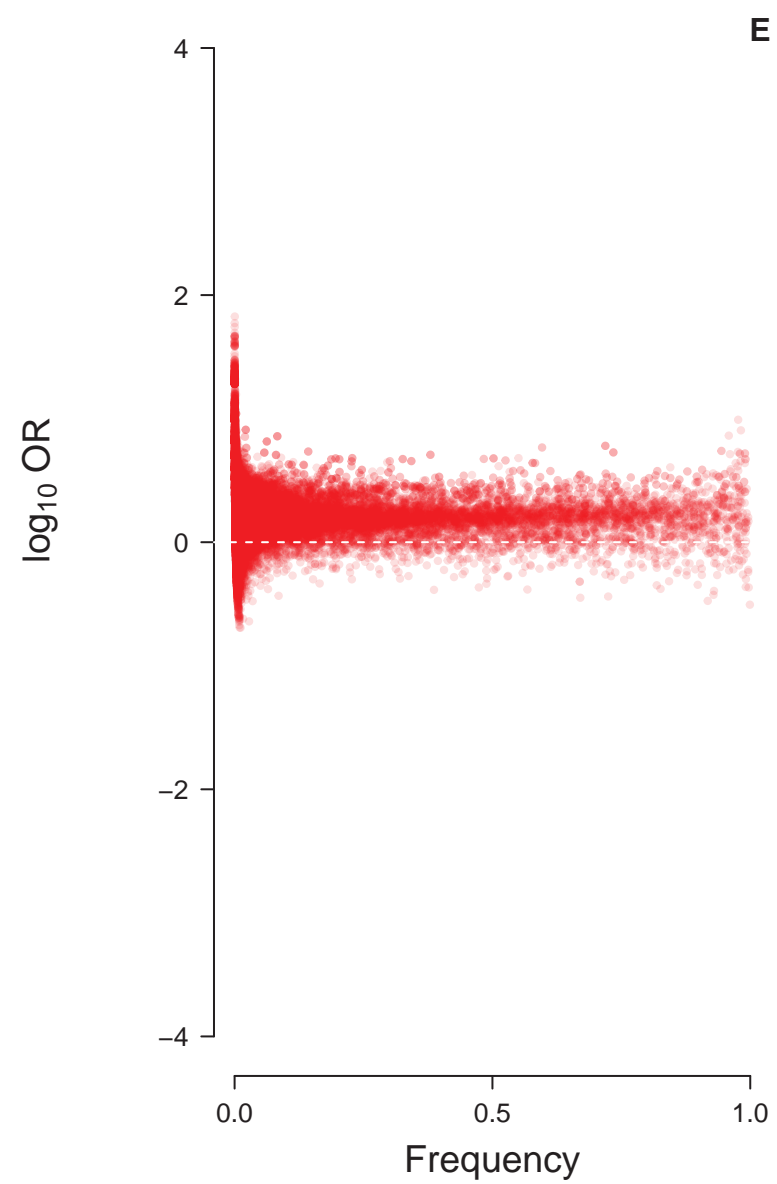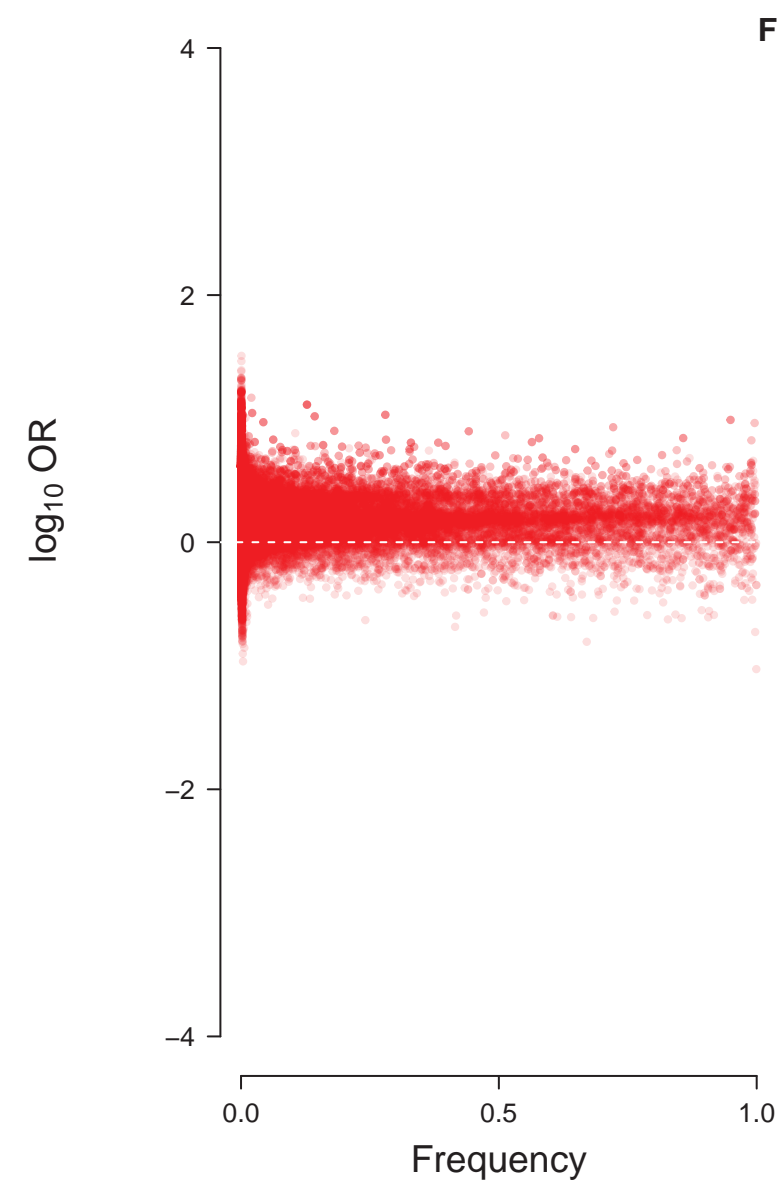

Supplement: S4 Fig — See legend to Fig 4 for details. (PDF) [file pone.0132150.s004.pdf]

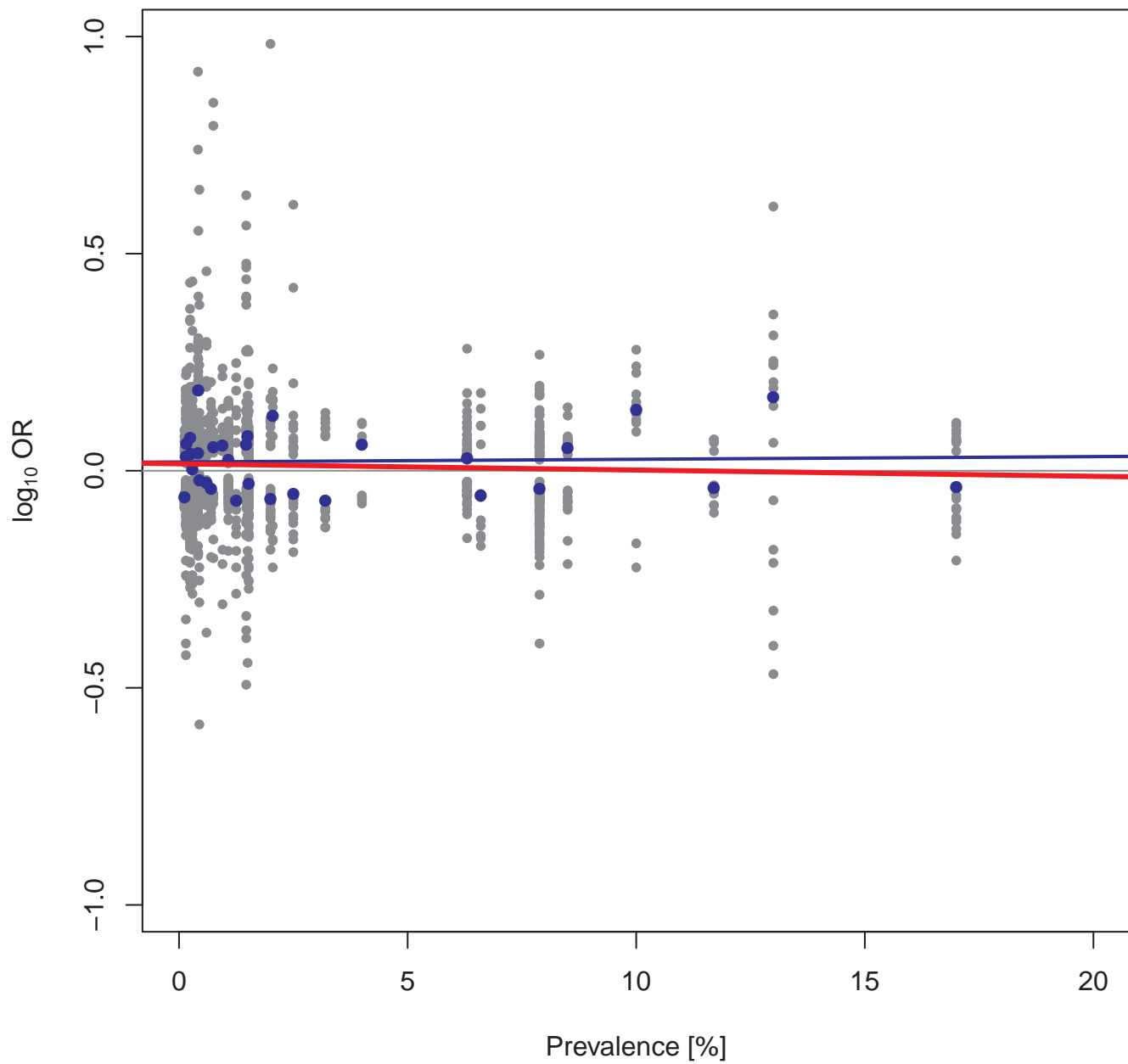

Supplement: S5 Fig — The analysis was based upon data for 31 traits taken from the GWAS catalogue. All ORs refer to the derived allele. Grey dots: trait-specific effect size (log10OR) in the GWAS catalogue for traits with reported prevalence between 0.1 and 20% (i.e. 1418 associations with p<5x10^-4^); blue dots: median log10OR per trait; red line: linear regression line for log10OR vs. trait prevalence (p = 0.38); blue line: linear regression line for median log10OR vs. trait prevalence (p = 0.71). (PDF) [file pone.0132150.s005.pdf]
