# Supplementary material for: Mutations Causing Complex Disease May under Certain Circumstances Be Protective in an Epidemiological Sense
Source: PLoS One. 2015 Jul 10;10(7):e0132150. doi: 10.1371/journal.pone.0132150 (PMC4498598; doi:10.1371/journal.pone.0132150)
Supplement: S1 Table — (PDF) [file pone.0132150.s008.pdf]

S1 Table: Merging of synonymous traits from the GWAS catalogue

Age-related macular degeneration, Age-related macular degeneration (CNV vs. GA), Age-related macular degeneration (CNV), Age-related macular degeneration (GA)
Alzheimer's disease, Alzheimer's disease (cognitive decline), Alzheimer's disease (late onset)
Atrial fibrillation, Atrial fibrillation/atrial flutter
Bladder cancer, Urinary bladder cancer
Breast cancer, Breast Cancer in BRCA1 mutation carriers, Estradiol plasma levels (breast cancer)
Esophageal cancer, Esophageal cancer (alcohol interaction), Esophageal cancer (squamous cell), Esophageal cancer and gastric cancer, Esophageal squamous cell cancer (length of survival), Upper aerodigestive tract cancers
Glioma, Glioma (high-grade)
Inflammatory bowel disease, Inflammatory bowel disease (early onset)
Lung adenocarcinoma, Lung cancer, Non-small cell lung cancer
Myocardial infarction, Myocardial infarction (early onset)
Ovarian cancer, Ovarian cancer in BRCA1 mutation carriers
Testicular germ cell cancer, Testicular germ cell tumor
Vitiligo, Vitiligo (non-segmental)
