# Supplementary material for: Mutations Causing Complex Disease May under Certain Circumstances Be Protective in an Epidemiological Sense
Source: PLoS One. 2015 Jul 10;10(7):e0132150. doi: 10.1371/journal.pone.0132150 (PMC4498598; doi:10.1371/journal.pone.0132150)
Supplement: S2 Table — (PDF) [file pone.0132150.s009.pdf]

S6 Table: Selected SNP associations identified from the GWAS catalogue

Disease / Trait	Prevalence estimate [%]	Number of associations reported*		Reference for prevalence estimate
		$p<5\times10^{-4}$	$p<5\times10^{-8}$	
Age-related macular degeneration<br>Age-related macular degeneration (CNV vs. GA)<br>Age-related macular degeneration (CNV)<br>Age-related macular degeneration (GA)	1.47[1]	101 (57)	64 (39)	[1]
Alzheimer's disease<br>Alzheimer's disease (cognitive decline)<br>Alzheimer's disease (late onset)	13.0[2]	90 (16)	39 (8)	Alzheimer's Association, Alzheimer's Disease Facts and Figures (2010): http://www.alz.org/documents_custom/report_alzfactsfigures2010.pdf
Asthma	8.5	37 (33)	28 (26)	National Health Interview Survey (NHIS) Data 2011: http://www.cdc.gov/asthma/nhis/2011/table4-1.htm
Atopic dermatitis	17	25 (23)	15 (15)	[2]
Atrial fibrillation<br>Atrial fibrillation/atrial flutter	0.95	18 (16)	16 (14)	[3]
Behcet's disease	0.0052	12 (9)	11 (8)	[4]
Bladder cancer<br>Urinary bladder cancer	0.1178	17 (13)	13 (10)	Surveillance, Epidemiology, and End Results (SEER) Program, Nov 2012 data submission: http://seer.cancer.gov/csr/1975_2010/
Breast cancer<br>Breast Cancer in BRCA1 mutation carriers<br>Estradiol plasma levels (breast cancer)	1.0793[3]	166 (129)	109 (99)	Surveillance, Epidemiology, and End Results (SEER) Program, Nov 2012 data submission: http://seer.cancer.gov/csr/1975_2010/
Celiac disease	0.75[4]	45 (30)	30 (19)	[5]

Chronic lymphocytic leukemia	0.0145	18 (14)	13 (11)	Surveillance, Epidemiology, and End Results (SEER) Program, Nov 2012 data submission: http://seer.cancer.gov/csr/1975_2010/
Colorectal cancer	0.2591	50 (26)	24 (20)	Surveillance, Epidemiology, and End Results (SEER) Program, Nov 2012 data submission: http://seer.cancer.gov/csr/1975_2010/
Coronary heart disease	6.3	72 (58)	52 (40)	Centers for Disease Control; Summary Health Statistics for US Adults: National Health Interview Survey (NHIS) 2011: http://www.cdc.gov/nchs/data/series/sr_10/sr10_256.pdf
Crohn's disease	0.15	185 (158)	160 (143)	[6]
Esophageal cancer<br>Esophageal cancer (alcohol interaction)<br>Esophageal cancer (squamous cell)<br>Esophageal cancer and gastric cancer<br>Esophageal squamous cell cancer (length of survival)<br>Upper aerodigestive tract cancers	0.0083	30 (22)	24 (19)	Surveillance, Epidemiology, and End Results (SEER) Program, Nov 2012 data submission: http://seer.cancer.gov/csr/1975_2010/
Glioma<br>Glioma (high-grade)	0.0264	15 (15)	13 (13)	Surveillance, Epidemiology, and End Results (SEER) Program, Nov 2012 data submission: http://seer.cancer.gov/csr/1975_2010/
Graves' disease	1.25[5]	24 (18)	22 (16)	[7]
Inflammatory bowel disease<br>Inflammatory bowel disease (early onset)	0.445[6]	127 (115)	126 (114)	[6]
Interstitial lung disease	0.07405[7]	16 (15)	11 (11)	[8]
Intracranial aneurysm	3.2	15 (15)	11 (11)	[9]
Lung cancer<br>Lung adenocarcinoma<br>Non-small cell lung cancer	0.0965	29 (26)	23 (22)	Surveillance, Epidemiology, and End Results (SEER) Program, Nov 2012 data submission: http://seer.cancer.gov/csr/1975_2010/
Melanoma	0.1623	20 (12)	15 (10)	Surveillance, Epidemiology, and End Results (SEER) Program, Nov 2012 data submission: http://seer.cancer.gov/csr/1975_2010/

Migraine	11.7	16 (10)	10 (6)	[10]
Multiple sclerosis	0.0765[8]	64 (48)	22 (18)	[11]
Myocardial infarction<br>Myocardial infarction (early onset)	4.0	10 (9)	10 (9)	Centers for Disease Control; Mobidity and Mortality Weekly report (2007): http://www.cdc.gov/mmwr/PDF/wk/mm5606.pdf
Ovarian cancer<br>Ovarian cancer in BRCA1 mutation carriers	0.063[3]	23 (8)	20 (7)	Surveillance, Epidemiology, and End Results (SEER) Program, Nov 2012 data submission: http://seer.cancer.gov/csr/1975_2010/
Paget's disease	2.05[9]	13 (11)	11 (9)	[12]
Parkinson's disease	2[10]	48 (37)	33 (22)	Alzheimer’s Association, Parkinson's Disease Dementia: http://www.alz.org/dementia/downloads/topicsheet_parkinsons.pdf
Polycystic ovary syndrome	6.6[3]	12 (12)	12 (12)	[13]
Primary biliary cirrhosis	0.05	33 (30)	30 (27)	[14]
Prostate cancer	1.5246[11]	93 (90)	80 (77)	Surveillance, Epidemiology, and End Results (SEER) Program, Nov 2012 data submission: http://seer.cancer.gov/csr/1975_2010/
Psoriasis	2.5[12]	32 (29)	23 (21)	National Psoriasis Foundation: https://www.psoriasis.org/about/stats
Restless legs syndrome	10.0	12 (12)	12 (12)	[15]
Rheumatoid arthritis	0.6	83 (64)	44 (36)	Centers for Disease Control: http://www.cdc.gov/arthritis/basics/rheumatoid.htm
Schizophrenia	0.7	51 (36)	16 (14)	World health Organization (WHO): http://www.who.int/mental_health/management/schizophrenia/en/
Sudden cardiac arrest	0.095[13]	51 (1)	13 (1)	[16]
Systemic lupus erythematosus	0.241[14]	92 (77)	55 (50)	[17]
Systemic sclerosis	0.0744	22 (8)	14 (5)	[18]
Testicular germ cell tumor<br>Testicular germ cell cancer	0.0724[7]	31 (28)	28 (25)	Surveillance, Epidemiology, and End Results (SEER) Program, Nov 2012 data submission: http://seer.cancer.gov/csr/1975_2010/
Type 1 diabetes	0.415[15]	63 (41)	43 (29)	Centers for Disease Control: http://www.cdc.gov/diabetes/pubs/factsheet11/fastfacts.htm

Type 2 diabetes	7.885[15]	157 (129)	91 (76)	Centers for Disease Control: http://www.cdc.gov/diabetes/pubs/factsheet11/fastfacts.htm
Ulcerative colitis	0.294	109 (100)	91 (85)	[6]
Venous thromboembolism	0.422	18 (15)	14 (11)	[19]
Vitiligo<br>Vitiligo (non-segmental)	1.5[16]	33 (27)	29 (25)	American Vitiligo Research Foubdation: http://www.avrf.org/facts/vitiligo_questions.htm
**43 traits**	**0.0 – 20.0**	**2178 (1642)**	**1520 (1245)**	
**31 traits**	**0.1 – 20.0**	**1834 (1418)**	**1298 (1078)**	
**30 traits**	**0.1 – 15.0**	**1809 (1395)**	**1283 (1063)**	

[*]Number of reported associations with reliable information on the evolutionary status of the risk allele variant are shown in brackets, respectively.

[1] prevalence in US population ≥ 40 years

[2] prevalence in US population ≥ 65 years

[3] prevalence among females only

[4] mid-point of reported prevalence range (US: 0.5-1.0%)

[5] mid-point of reported prevalence range (Caucasian: 0.5-2.0%)

[6] sum of prevalence of Crohn disease and ulcerative colitis

[7] sex average (men: 0.0809%; women: 0.0672%)

[8] mid-point of reported prevalence range (US: 0.058-0.095%)

[9] sex average (men: 2.5%; women: 1.6%).

[10] prevalence in adult population ≥ 65 years.

[11] prevalence among males only

[12] mid-point of reported prevalence range (worldwide: 2.0-3.0%).

[13] age- and sex-adjusted incidence rate

[14] prevalence in US population ≥ 17 years.

[15] Some 8.3% of the US population ≥ 20 years have diabetes, with type 2 diabetes accounting for 95% of all diagnosed cases.

[16] mid-point of reported prevalence range (1.0-2.0%).

References

1. Friedman DS, O'Colmain BJ, Munoz B, Tomany SC, McCarty C, et al. (2004) Prevalence of age-related macular degeneration in the United States. Arch Ophthalmol 122: 564-572.
2. Piliang M (2010) Atopic Dermatitis. In: Carey WD, editor. Current Clinical Medicine. 2nd ed: Sounders WB Company.
3. Go AS, Hylek EM, Phillips KA, Chang Y, Henault LE, et al. (2001) Prevalence of diagnosed atrial fibrillation in adults: national implications for rhythm management and stroke prevention: the AnTicoagulation and Risk Factors in Atrial Fibrillation (ATRIA) Study. JAMA 285: 2370-2375.
4. Calamia KT, Wilson FC, Icen M, Crowson CS, Gabriel SE, et al. (2009) Epidemiology and clinical characteristics of Behcet's disease in the US: a population-based study. Arthritis Rheum 61: 600-604.
5. Brown AC (2012) Gluten sensitivity: problems of an emerging condition separate from celiac disease. Expert Rev Gastroenterol Hepatol 6: 43-55.
6. Jacobsen BA, Fallingborg J, Rasmussen HH, Nielsen KR, Drewes AM, et al. (2006) Increase in incidence and prevalence of inflammatory bowel disease in northern Denmark: a population-based study, 1978-2002. Eur J Gastroenterol Hepatol 18: 601-606.
7. Ploski R, Szymanski K, Bednarczuk T (2011) The genetic basis of graves' disease. Curr Genomics 12: 542-563.
8. Coultas DB, Zumwalt RE, Black WC, Sobonya RE (1994) The epidemiology of interstitial lung diseases. Am J Respir Crit Care Med 150: 967-972.
9. Vlak MH, Algra A, Brandenburg R, Rinkel GJ (2011) Prevalence of unruptured intracranial aneurysms, with emphasis on sex, age, comorbidity, country, and time period: a systematic review and meta-analysis. Lancet Neurol 10: 626-636.
10. Lipton RB, Bigal ME, Diamond M, Freitag F, Reed ML, et al. (2007) Migraine prevalence, disease burden, and the need for preventive therapy. Neurology 68: 343-349.
11. Noonan CW, Williamson DM, Henry JP, Indian R, Lynch SG, et al. (2010) The prevalence of multiple sclerosis in 3 US communities. Preventing Chronic Disease 7: A12.
12. Cooper C, Harvey NC, Dennison EM, van Staa TP (2006) Update on the epidemiology of Paget's disease of bone. J Bone Miner Res 21 Suppl 2: P3-8.
13. Azziz R, Woods KS, Reyna R, Key TJ, Knochenhauer ES, et al. (2004) The prevalence and features of the polycystic ovary syndrome in an unselected population. J Clin Endocrinol Metab 89: 2745-2749.
14. Liu X, Invernizzi P, Lu Y, Kosoy R, Bianchi I, et al. (2010) Genome-wide meta-analyses identify three loci associated with primary biliary cirrhosis. Nature Genetics 42: 658-660.
15. Phillips B, Hening W, Britz P, Mannino D (2006) Prevalence and correlates of restless legs syndrome: results from the 2005 National Sleep Foundation Poll. Chest 129: 76-80.
16. Nichol G, Thomas E, Callaway CW, Hedges J, Powell JL, et al. (2008) Regional variation in out-of-hospital cardiac arrest incidence and outcome. JAMA 300: 1423-1431.
17. Ward MM (2004) Prevalence of physician-diagnosed systemic lupus erythematosus in the United States: results from the third national health and nutrition examination survey. J Womens Health (Larchmt) 13: 713-718.

18. Bernatsky S, Joseph L, Pineau CA, Belisle P, Hudson M, et al. (2009) Scleroderma prevalence: demographic variations in a population-based sample. Arthritis Rheum 61: 400-404.
19. Deitelzweig SB, Johnson BH, Lin J, Schulman KL (2011) Prevalence of clinical venous thromboembolism in the USA: current trends and future projections. Am J Hematol 86: 217-220.
