# Supplementary material for: Mutations Causing Complex Disease May under Certain Circumstances Be Protective in an Epidemiological Sense
Source: PLoS One. 2015 Jul 10;10(7):e0132150. doi: 10.1371/journal.pone.0132150 (PMC4498598; doi:10.1371/journal.pone.0132150)
Supplement: S3 Table — (PDF) [file pone.0132150.s010.pdf]

S3 Table: Mutational spectrum of five unlinked loci under two different penetrance models

Model parameters	Median number of mutations (IQR)		
	Per population	Per case	Per control
**Rare disease (prevalence: 0.1-1%)**			
**Multiplicative**			
γ=0.3	3 (3-5)	1.000 (1.000-1.000)	0.012 (0.006-0.019)
γ=0.1	4 (3-7)	1.011 (1.000-1.034)	0.055 (0.028-0.075)
**Logistic**			
α=-5; *β*=1	5 (3-8)	0.384 (0.174-0.546)	0.166 (0.069-0.260)
α=-5; *β*=0.5	9 (5-13)	0.859 (0.629-1.043)	0.627 (0.449-0.789)
**Common disease (prevalence: 1-5%)**			
**Multiplicative**			
γ=0.3	5 (3-8)	1.027 (1.007-1.055)	0.080 (0.049-0.105)
γ=0.1	8 (5-12)	1.152 (1.062-1.260)	0.361 (0.251-0.432)
**Logistic**			
α=-5; *β*=1	17.50 (12-24)	2.298 (2.062-2.477)	1.435 (1.251-1.594)
α=-5; *β*=0.5	39 (30-48)	4.389 (4.163-4.627)	3.467 (3.260-3.645)
**Pandemic disease (prevalence: 10-20%)**			
**Multiplicative**			
γ=0.3	10 (6-14.25)	1.258 (1.143-1.372)	0.453 (0.370-0.512)
γ=0.1	22 (16-30)	2.429 (2.216-2.633)	1.879 (1.696-1.991)
**Logistic**			
α=-5; *β*=1	34 (26-42.25)	4.091 (3.852-4.299)	2.768 (2.578-2.942)
α=-5; *β*=0.5	69 (57-85)	7.780 (7.550-8.010)	6.280 (6.035-6.471)

IQR: inter-quartile range
