# Supplementary material for: Mutations Causing Complex Disease May under Certain Circumstances Be Protective in an Epidemiological Sense
Source: PLoS One. 2015 Jul 10;10(7):e0132150. doi: 10.1371/journal.pone.0132150 (PMC4498598; doi:10.1371/journal.pone.0132150)
Supplement: S4 Table — (PDF) [file pone.0132150.s011.pdf]

S4 Table: Proportion of epidemiologically protective mutations in five unlinked loci under two different penetrance models

Model parameters	Percentage mutations with OR<1.0	Percentage mutations with OR>m or OR<1/m (percentage of these with OR<1)		Percentage mutations with OR<1.0 among those with frequency <f		
		m=1.5	m=2.0	f=0.001	f=0.01	f=0.05
**Rare disease (prevalence: 0.1-1%)**						
**Multiplicative**						
γ=0.3	0.00	100.0 (0.00)	100.0 (0.00)	0.00	0.00	0.00
γ=0.1	0.00	100.0 (0.00)	100.00 (0.00)	0.00	0.00	0.00
**Logistic**						
α=-5; β=1	2.37	92.28 (0.82)	80.49 (0.28)	0.00	0.00	3.40
α=-5; β=0.5	9.95	69.60 (3.97)	42.55 (2.48)	0.00	0.00	11.91
**Common disease (prevalence: 1-5%)**						
**Multiplicative**						
γ=0.3	0.02	99.98 (0.00)	99.85 (0.00)	0.00	0.04	0.02
γ=0.1	0.50	98.03 (0.09)	94.75 (0.02)	0.00	1.04	0.70
**Logistic**						
α=-5; β=1	5.31	85.90 (1.69)	71.43 (0.90)	0.00	5.82	5.40
α=-5; β=0.5	14.62	64.13 (7.00)	38.10 (4.75)	0.00	14.97	15.35
**Pandemic disease (prevalence: 10-20%)**						
**Multiplicative**						
γ=0.3	1.00	96.90 (0.28)	93.05 (0.15)	2.79	1.97	1.38
γ=0.1	9.70	66.47 (4.26)	33.23 (3.62)	11.88	15.56	11.95
**Logistic**						
α=-5; β=1	9.77	78.07 (4.46)	58.28 (2.93)	8.57	10.64	9.50
α=-5; β=0.5	19.07	58.97 (11.02)	35.65 (8.18)	12.14	20.25	18.82

OR: odds ratio
