# Supplementary material for: Mutations Causing Complex Disease May under Certain Circumstances Be Protective in an Epidemiological Sense
Source: PLoS One. 2015 Jul 10;10(7):e0132150. doi: 10.1371/journal.pone.0132150 (PMC4498598; doi:10.1371/journal.pone.0132150)
Supplement: S5 Table — (PDF) [file pone.0132150.s012.pdf]

S5 Table: Mutational spectrum of ten unlinked loci under two different penetrance models

Model parameters	Median number of mutations (IQR)		
	Per population	Per case	Per control
**Rare disease (prevalence: 0.1-1%)**			
**Multiplicative**			
γ=0.3	3<br>(2-5)	1.000<br>(1.000-1.010)	0.012<br>(0.006-0.018)
γ=0.1	5<br>(3-7)	1.015<br>(1.000-1.040)	0.054<br>(0.032-0.075)
**Logistic**			
α=-5; *β*=1	5<br>(3-8)	0.387<br>(0.181-0.546)	0.173<br>(0.073-0.257)
α=-5; *β*=0.5	9<br>(5-14)	0.847<br>(0.632-1.051)	0.631<br>(0.445-0.780)
**Common disease (prevalence: 1-5%)**			
**Multiplicative**			
γ=0.3	5<br>(4-8)	1.030<br>(1.010-1.058)	0.077<br>(0.049-0.104)
γ=0.1	8<br>(5-12)	1.157<br>(1.067-1.252)	0.349<br>(0.239-0.429)
**Logistic**			
α=-5; *β*=1	17<br>(13-24)	2.339<br>(2.108-2.515)	1.446<br>(1.276-1.586)
α=-5; *β*=0.5	38<br>(30-48)	4.432<br>(4.201-4.613)	3.469<br>(3.274-3.636)
**Pandemic disease (prevalence: 10-20%)**			
**Multiplicative**			
γ=0.3	10<br>(6-14)	1.266<br>(1.148-1.374)	0.457<br>(0.372-0.515)
γ=0.1	22<br>(16-30)	2.434<br>(2.249-2.626)	1.884<br>(1.684-1.991)
**Logistic**			
α=-5; *β*=1	33<br>(26-42)	4.115<br>(3.924-4.301)	2.751<br>(2.572-2.908)
α=-5; *β*=0.5	70<br>(58-83)	7.784<br>(7.596-7.994)	6.230<br>(6.042-6.424)

IQR: inter-quartile range
