# Supplementary material for: Mutations Causing Complex Disease May under Certain Circumstances Be Protective in an Epidemiological Sense
Source: PLoS One. 2015 Jul 10;10(7):e0132150. doi: 10.1371/journal.pone.0132150 (PMC4498598; doi:10.1371/journal.pone.0132150)
Supplement: S6 Table — (PDF) [file pone.0132150.s013.pdf]

S6 Table: Proportion of epidemiologically protective mutations in ten unlinked loci under two different penetrance models

Model parameters	Percentage mutations with OR<1.0	Percentage mutations with OR>*m* or OR<1/*m* (percentage of these with OR<1)		Percentage mutations with OR<1.0 among those with frequency <*f*		
		m=1.5	*m*=2.0	*f*=0.001	*f*=0.01	*f*=0.05
		**Rare disease (prevalence: 0.1-1%)**				
**Multiplicative**						
γ=0.3	0.00	100.0 (0.00)	100.0 (0.00)	0.00	0.00	0.00
γ=0.1	0.00	100.0 (0.00)	100.00 (0.00)	0.00	0.00	0.00
**Logistic**						
α=-5; *β*=1	2.49	92.57 (0.66)	80.72 (0.24)	0.00	0.00	3.56
α=-5; *β*=0.5	9.63	70.72 (3.83)	42.86 (2.36)	0.00	0.00	11.62
		**Common disease (prevalence: 1-5%)**				
**Multiplicative**						
γ=0.3	0.00	99.98 (0.00)	99.94 (0.00)	0.00	0.00	0.00
γ=0.1	0.48	98.32 (0.06)	95.73 (0.00)	0.00	1.00	0.66
**Logistic**						
α=-5; *β*=1	4.11	89.16 (1.19)	75.41 (0.59)	0.00	6.17	4.85
α=-5; *β*=0.5	10.80	64.47 (4.55)	33.98 (3.41)	0.00	14.18	13.26
		**Pandemic disease (prevalence: 10-20%)**				
**Multiplicative**						
γ=0.3	0.89	97.08 (0.26)	94.05 (0.03)	3.07	1.77	1.23
γ=0.1	7.76	69.84 (3.84)	29.88 (3.12)	11.48	13.98	10.35
**Logistic**						
α=-5; *β*=1	6.35	82.63 (2.35)	61.58 (1.45)	8.11	8.91	6.93
α=-5; *β*=0.5	13.72	57.92 (6.92)	30.77 (5.12)	12.19	18.16	15.09

OR: odds ratio
